# An assessment of the carbon stocks and sodicity tolerance of disturbed *Melaleuca* forests in Southern Vietnam

**DOI:** 10.1186/s13021-015-0025-6

**Published:** 2015-07-14

**Authors:** Da B Tran, Tho V Hoang, Paul Dargusch

**Affiliations:** 1 The Vietnam Forestry University, Hanoi, Vietnam; 2 School of Geography, Planning and Environmental Management, The University of Queensland, Brisbane, QLD Australia

**Keywords:** Carbon sequestration, Climate change, *Melaleuca*, REED+, Sodicity

## Abstract

**Background:**

In the lower Mekong Basin and coastal zones of Southern Vietnam, forests dominated by the genus *Melaleuca* have two notable features: most have been substantially disturbed by human activity and can now be considered as degraded forests; and most are subject to acute pressures from climate change, particularly in regards to changes in the hydrological and sodicity properties of forest soil.

**Results:**

Data was collected and analyzed from five typical *Melaleuca* stands including: (1) primary *Melaleuca* forests on sandy soil (VS1); (2) regenerating *Melaleuca* forests on sandy soil (VS2); (3) degraded secondary *Melaleuca* forests on clay soil with peat (VS3); (4) regenerating *Melaleuca* forests on clay soil with peat (VS4); and (5) regenerating *Melaleuca* forests on clay soil without peat (VS5). Carbon densities of VS1, VS2, VS3, VS4, and VS5 were found to be 275.98, 159.36, 784.68, 544.28, and 246.96 tC/ha, respectively. The exchangeable sodium percentage of *Melaleuca* forests on sandy soil showed high sodicity, while those on clay soil varied from low to moderate sodicity.

**Conclusions:**

This paper presents the results of an assessment of the carbon stocks and sodicity tolerance of natural *Melaleuca cajuputi* communities in Southern Vietnam, in order to gather better information to support the improved management of forests in the region. The results provide important information for the future sustainable management of *Melaleuca* forests in Vietnam, particularly in regards to forest carbon conservation initiatives and the potential of *Melaleuca* species for reforestation initiatives on degraded sites with highly sodic soils.

**Electronic supplementary material:**

The online version of this article (doi:10.1186/s13021-015-0025-6) contains supplementary material, which is available to authorized users.

## Background

Numerous studies have shown that tropical wetlands typically contain large carbon stocks [1–7]. Protecting and restoring tropical coastal wetlands is considered a critical part of how society adapts to and mitigates global climate change [8].

Large areas of *Melaleuca* forests in Vietnam are disturbed ecosystems that experience extreme conditions, and are associated with floods and/or sodic soils. They mostly occur in the lower Mekong Basin, which has been severely impacted by climate change [9–12]. Little is known about the carbon sequestration potential of disturbed *Melaleuca* forests in Australasia and South-East Asia where the genus occurs. Carbon stocks of *Melaleuca* forests are generally considered to be low (i.e. about 27.8 tC/ha estimated by Australian Government Office [13]). However, Tran et al. [14] suggested that this has been grossly under-estimated and that *Melaleuca cajuputi* forests on peatland soils in Vietnam, Indonesia and Malaysia are likely to have a high potential for carbon sequestration.

Sea level rise has significant impacts on the coastal zone, where soils will become saline and/or highly sodic [15]. Sodic soils are distinguished by an excessively high concentration of Sodium (Na) in their cation exchange complex. High sodicity causes soil instability due to poor physical and chemical properties, which affects plant growth and can have a more significant impact than excessive salinity growth [16, 17]. Sodicity impacts plant growth in three ways, including: soil dispersion, specific ion effects, and nutritional imbalance in plants [18, 19]. Excessive sodium concentrations cause clay dispersion which is the primary physical effect of the sodic soil. Sodium-induced dispersion can reduce water infiltration, decrease hydraulic conductivity, and increase soil surface crusting that strongly affect roots such as root penetration, root development, and blocking plant uptake of moisture and nutrients [19].

Except for those containing mangroves and other halophytes, most ecosystems are severely affected by salinity and/or sodicity. A few studies have examined saline-sodic soils in shrimp farming areas in the coastal regions of Vietnam (i.e. ECe = 29.25 dS/m and exchangeable sodium percentage ranged from 9.63 to 72.07%, which had a big impact on plant cultivation systems [20]).

Several studies (such as Dunn et al. [21], Niknam and McComb [22], van der Moezel et al. [23, 24]) have examined the tolerance of woody species such as *Acacia, Eucalyptus, Melaleuca,* and *Casuarina* species to salinity and/or sodicity, but more research is required. This paper examines the carbon stocks of disturbed *Melaleuca* forests and the sodicity tolerance of *M. cajuputi* forests in Southern Vietnam.

## Results and discussion

### Characteristics of the typical *Melaleuca* forests in the study areas

The major characteristics of five *Melaleuca* forests types examined include standing trees, an understory, and saturated conditions (Table 1). The variation in these characteristics not only distinguishes the different stands but also improves understanding of their carbon stocks.

**Table 1:** Major characteristics of five typical *Melaleuca* forests in the study areas

Forest types	Tree classes	Code	Stand trees								Understory	Saturation levels
			*Density* (trees/ha)		*DBH* (cm)		*BA* (m^2^/ha)		*Height* (m)			
			Mean	SE	Mean	SE	Mean	SE	Mean	SE		
Primary *Melaleuca* on sandy soil	DBH < 5 cm	VS1C0	800	248.3	3.87	0.11	na	na	6.00	0.28	*Leptocarpus sp.* *Lepironia sp.* *Hanguana sp.* *Eleocharis sp.* *Euriocaulon sp.* *Xyris sp.* *Stenochlaena sp.* *Melastoma sp.* *Imperata sp.*	Including non-inundated, seasonal, and permanent inundation
	5 cm ≤ DBH < 10 cm	VS1C1	400	100.0	7.18	0.36	na	na	9.81	0.68		
	10 cm ≤ DBH < 20 cm	VS1C2	750	273.8	14.63	0.22	na	na	14.80	0.26		
	20 cm ≤ DBH < 30 cm	VS1C3	285	34.0	24.33	0.49	na	na	18.44	0.40		
	30 cm ≤ DBH < 40 cm	VS1C4	80	28.3	34.37	0.90	na	na	20.17	0.97		
	DBH ≥ 40 cm	VS1C5	20	10.0	48.73	3.75	na	na	22.20	1.77		
	All classes	VS1	2,330	558.0	16.71	0.55	41.54	6.16	14.69	0.30		
Regenerating *Melaleuca* on sandy soil	DBH < 5 cm	VS2C0	5,450	2,850.0	3.63	0.07	na	na	6.13	0.16		Including non-inundated and seasonal inundation
	5 cm ≤ DBH < 10 cm	VS2C1	5,500	700.0	7.07	0.14	na	na	8.08	0.15		
	DBH ≥ 10 cm	na	na	na	na	na	na	na	na	na		
	All classes	VS2	10,950	3,550.0	5.36	0.14	28.41	3.14	7.11	0.13		
Degraded secondary *Melaleuca* on clay soil with peat	DBH < 5 cm	VS3C0	150	na	4.41	0.23	na	na	5.00	0.29	*Stenochlaenapalustris* *Phragmitesvallatoria* *Melastomadodecandrum* *Diplaziumesculentum* *Lygodiumscandens* *Aspleniumnidus* *Scleriasumatrensis* *Cassia tora* *Paederiafoetida* *Flagellariaindica* *Cayratiatrifolia*	Including seasonal and permanent inundation
	5 cm ≤ DBH < 10 cm	VS3C1	350	na	7.12	0.68	na	na	4.57	0.38		
	10 cm ≤ DBH < 20 cm	VS3C2	440	20.0	13.11	0.36	na	na	10.44	0.41		
	20 cm ≤ DBH < 30 cm	VS3C3	30	na	25.00	1.20	na	na	14.33	0.17		
	30 cm ≤ DBH < 40 cm	VS3C4	10	na	35.35	na	na	na	12.50	na		
	DBH ≥ 40 cm	VS3C5	na	na	na	na	na	na	na	na		
	All classes	VS3	980	560.0	12.93	0.71	10.29	4.74	9.69	0.45		
Regenerating *Melaleuca* on clay soil with peat	DBH < 5 cm	VS4C0	3,867	2,258.6	3.84	0.06	na	na	4.15	0.11		Including seasonal and permanent inundation
	5 cm ≤ DBH < 10 cm	VS4C1	5,967	176.4	7.20	0.12	na	na	6.68	0.17		
	DBH ≥ 10 cm	na	na	na	na	na	na	na	na	na		
	All classes	VS4	9,833	2,265.9	5.88	0.12	30.14	1.46	5.68	0.13		
Regenerating *Melaleuca* on clay soil without peat	DBH < 5 cm	VS5C0	2,133	592.6	3.82	0.09	na	na	4.95	0.17		Including seasonal and permanent inundation
	5 cm ≤ DBH < 10 cm	VS5C1	4,733	1,560.3	7.27	0.13	na	na	8.65	0.31		
	DBH ≥ 10 cm	na	na	na	na	na	na	na	na	na		
	All classes	VS5	6,867	1,970.1	6.20	0.14	23.02	8.53	7.50	0.25		

**Figure 1:**
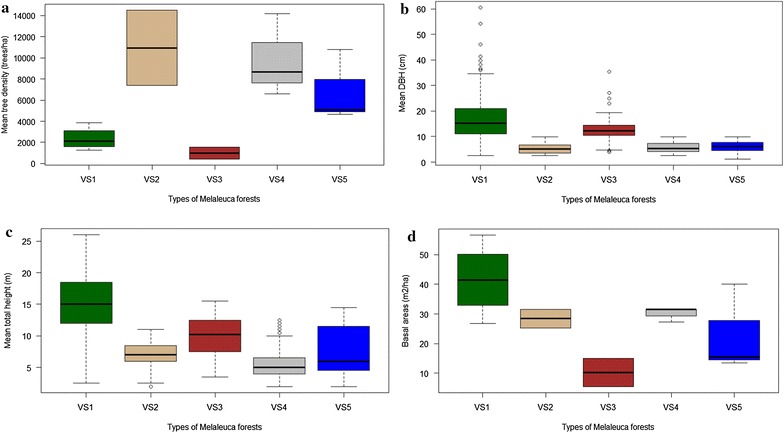
Traits of five *Melaleuca* forest types in the study areas: **a** stand densities, **b** diameter at bread height, **c** total height, and **d** basal areas.

The stand densities of the five typical *Melaleuca* forest types varied considerably: they were 2,330, 10,950, 980, 9,833, and 6,867 trees/ha for VS1, VS2, VS3, VS4, and VS5, respectively (Table 1). Within each study site, the tree densities of regenerating forests (VS2, VS4, and VS5) were significantly higher than primary forests (VS1) and secondary forests (VS2) (Figure 1a). The increased stand densities of types VS2, VS4, and VS5 were mostly comprised of trees with a diameter at breast height (DBH) <10 cm. In contrast, VS1 was dominated by trees with DBH < 20 cm (accounting for 84.3%), with the balance of trees having a DBH ≥ 20 cm (including 4.2% of trees with DBH ≥ 30), while VS3 was mostly dominated by trees with a 5 cm ≤ DBH < 20 cm (accounting for 96%), with the balance having a 20 cm ≤ DBH < 40 cm (accounting for 4%) (Table 1).

Average DBH of all stand classes were 16.71, 5.36, 12.93, 5.88, and 6.20 for VS1, VS2, VS3, VS4, and VS5, respectively (Figure 1b). There was a significant difference in DBH in the five *Melaleuca* forest types (χ^2^ = 446.86, *p* = 2.2e^−16^). However, post hoc test shows that there is no significant difference in tree DBH between VS1 and VS3, and between VS2, VS4, and VS5 (Additional file 1: 2b).

Average total height of all stand classes were 14.69, 7.11, 9.69, 5.68, and 7.50 m for VS1, VS2, VS3, VS4, and VS5, respectively (Figure 1c). There was a significant difference in the total height of the five *Melaleuca* forest types (χ^2^ = 11.616, *p* = 0.0088) (Additional file 1: 2c). Furthermore, the tree density of the five forest types was generally very high, especially of VS2, VS4 and VS5 (over 2,000 individuals/ha), which can contribute to a large biomass. The basal areas shown in Figure 1d further confirm the potential high biomass of VS2, VS4 and VS5 (BA = 28.41, 30.14, and 23.14 m^2^/ha, respectively). Furthermore, the basal area of VS1 is significantly greater than VS3, accounting for 41.45 and 10.29 m^2^/ha, respectively (F = 3.341, *p* = 0.0423) (Additional file 1: 2d).

Different species were found in the understorey of the various *Melaleuca* forest types. Key species for VS1 and VS2 include *Leptocarpus* sp., *Lepironia* sp., *Hanguana* sp., *Eleocharis* sp., *Euriocaulon* sp., *Xyris* sp., *Stenochlaena* sp., *Melastoma* sp., and *Imperata cylindrica.* For VS3, VS4, VS5, the following species dominate the understorey: *Stenochlaenapalustris* sp., *Phragmitesvallatoria* sp., *Melastomadodecandrum* sp., *Diplaziumesculentum* sp.*, Lygodiumscandens* sp., *Aspleniumnidus* sp., *Scleriasumatrensis, Cassia tora, Paederiafoetida* sp., *Flagellariaindica* sp., and *Cayratiatrifolia* sp. (Table 1).

### Carbon stocks of *Melaleuca* forests

**Figure 2:**
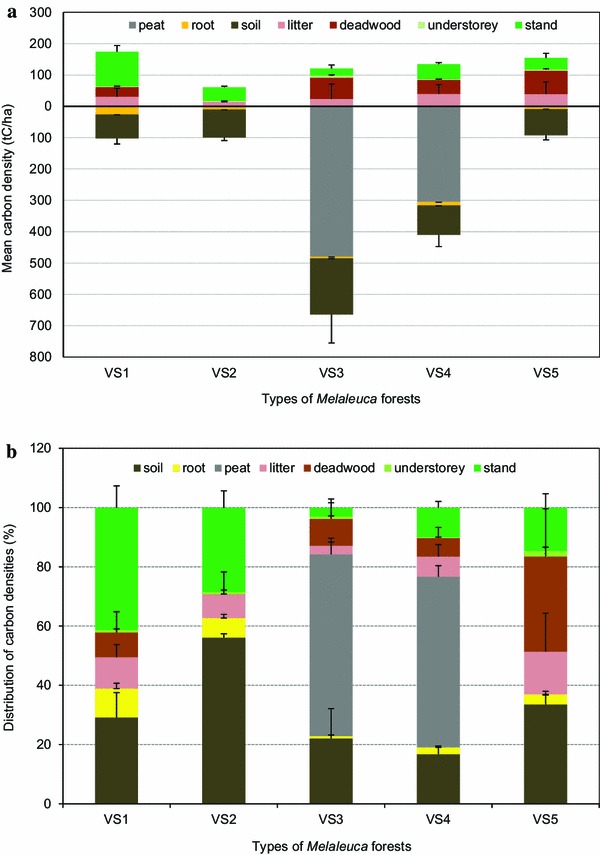
Carbon densities of five typical *Melaleuca* forests in the study areas: **a** mean carbon density, and **b** distribution of carbon densities.

The carbon densities of five typical *Melaleuca* forests in Southern Vietnam were 275.98, 159.36, 784.68, 544.28, and 246.96 tC/ha, respectively, for primary *Melaleuca* forests on sandy soil (VS1), regenerating *Melaleuca* forests on sandy soil (VS2), degraded secondary *Melaleuca* forests on clay soil with peat (VS3), regenerating *Melaleuca* forests on clay soil with peat (VS4), and regenerating *Melaleuca* forests on clay soil without peat (VS5) (Figure 2a). There is significant difference in carbon densities between the forest types (χ^2^ = 10.419, *p* = 0.0339) (Additional file 1: 2e). On sandy soils, the carbon density of VS1 was significantly greater (1.7 times) than VS2. The carbon density of *Melaleuca* forests on clay soil with peat was still high after disturbance (VS3 was 1.4 times higher than VS4). The carbon density of VS5 was lower than VS3 and VS4 because there was no peat layer.

On sandy soil, the stands and soil layers were the highest contributors to carbon density of VS1 (accounting for 41.34 and 29.11%, respectively), while VS2 has a high contribution from the soil layer, then stands (soil and stand categories contribute for carbon density of 56.15 and 28.53%, respectively) (Figure 2b). However, in the peat land, the greatest contribution of carbon densities for VS3 and VS4 are the peat and soil categories (accounting for 61.41%, 22.10% of VS3, and 57.66, and 16.72% of VS4, respectively). Separately, carbon density of VS5 is mostly linked to the soil, deadwood, and stand categories (accounting for 33.54, 32.16, and 14.66%, respectively) (Figure 2b).

### Variability of carbon stocks in different types of *Melaleuca* forests

**Figure 3:**
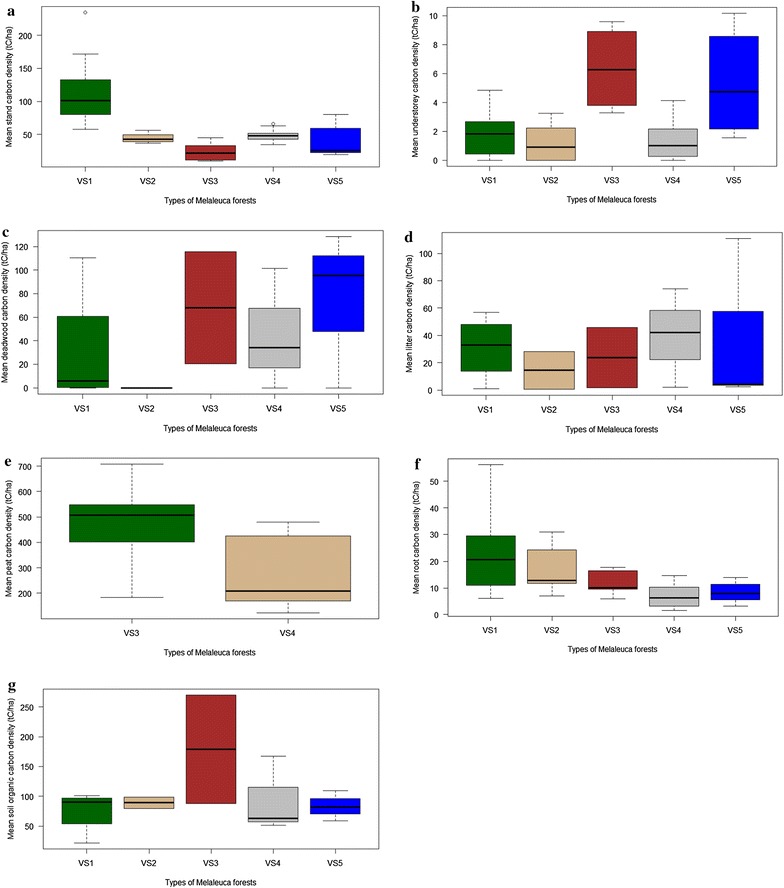
Carbon densities of carbon stock categories of five *Melaleuca* forests types in the study areas: **a** mean stand carbon density, **b** mean understorey carbon density, **c** mean deadwood carbon density, **d** mean litter carbon density, **e** mean peat carbon density, **f** mean root carbon density, and **g** mean soil organic carbon density.

This study investigated the carbon stocks of six categories: stands, understory, deadwood, litter, root, and soil for five types of *Melaleuca* forests in Southern Vietnam (Figure 3).

The carbon densities of stands of the various forest types were 110.67, 44.27, 22.79, 48.25, and 37.20 tC/ha for VS1, VS2, VS3, VS4, and VS5, respectively (Figure 3a). There was a significant difference in stand carbon density between the forest types (χ^2^ = 48.3184, *p* = 8.1e^−10^) (Additional file 1: 2f). The carbon density of the stand VS1 is the highest and is 2.5, 4.9, 2.3, and 3.0 times higher than VS2, VS3, VS4, and VS5. Surprisingly, there is no statistical difference in stand carbon densities between secondary forests (VS3) and regenerating forests (VS2, VS4 and VS5) (Additional file 1: 2f). These carbon stocks were lower those from other studies of different forests (e.g. 144 tC/ha for Asian tropical forests [25]; 200.23 tC/ha and 92.34 tC/ha of primary and secondary swamp forests in Indonesia (involving *Melaleuca* vegetation), respectively [26]).

The carbon densities of the understory in the *Melaleuca* forests of Vietnam were 2.45, 2.48, 6.23, 1.65, and 5.27 tC/ha for VS1, VS2, VS3, VS4, and VS5, respectively (Figure 3b). There was a statistically significant difference in understory carbon density between the forest types (χ^2^ = 30.7189, *p* = 3.49e^−6^) (Additional file 1: 2g). However, there was no significant difference in understory carbon density between *Melaleuca* forest types on sandy soils (VS1 and VS2). On clay soils, the understory carbon densities of VS3 and VS5 were significantly higher than VS4.

The carbon densities of deadwood in the forest types were 30.47, 0, 67.90, 45.06, and 74.59 tC/ha for VS1, VS2, VS3, VS4, and VS5, respectively (Figure 3c). There was a statistically significant difference in deadwood carbon density between the *Melaleuca* forest types (χ^2^ = 3.0978, *p* = 0.5416), but pairwise comparisons show no significant differences (Additional file 1: 2 h). Surprisingly, deadwood was not present in regenerating forests in the study sites on Phu Quoc Island. This is probably due to frequent forests fires and/or fuelwood collection by people associated crop cultivation.

Some of the carbon stock of *Melaleuca* forests is contributed by layers of coarse and fine litter. The carbon densities of the total litter layer of the forest types were 31.03, 14.45, 23.76, 57.35, and 39.23 tC/ha for VS1, VS2, VS3, VS4, and VS, respectively (Figure 3d). There was a statistically significant difference in overall litter carbon density between these forest types (χ^2^ = 1.5619, *p* = 0.08156), but pairwise comparisons show no significant differences (Additional file 1: 2i).

The carbon densities from peat of the *Melaleuca* forests were 479.62 and 294.57 tC/ha for secondary forests (VS3) and regenerating forests (VS4), respectively (Figure 3e). The carbon density from peat of VS3 is significantly greater than that of VS4 (χ^2^ = 5.2359, *p* = 0.0221) (Additional file 1: 2j). This is almost certainly due to peat being partly burned in the regenerating forest by the severe fire of 2002. In U Minh Thuong National Park, peat comprises the top soil layer, with a deep layer of clay below. The depth of the peat layer ranged from 15 to 62 cm in 18 soil cores, and the peat bulk density ranged from 0.19 to 0.3. The depths of the peat layer in this study were much thinner than in other forests (i.e. primary peat layer in U Minh Thuong was over 90 cm depth [27], and the thick peat layer in U Minh Ha was over 120 cm depth [28]).

The carbon densities of roots in the *Melaleuca* forests were 22.75, 16.97, 11.97, 6.99, and 8.35 tC/ha for VS1, VS2, VS3, VS4, and VS5, respectively (Figure 3f). There was a statistically significant difference in root carbon density between the forest types (χ^2^ = 22.437, *p* = 0.00016). The carbon densities of roots in *Melaleuca* forests in sandy soil were higher than those in clay soil, in particular, the root carbon density of VS2 was significant higher than that of VS4 (Additional file 1: 2k).

Organic soil carbon densities to a 30 cm depth in the study areas were 75.81, 89.22, 178.93, 93.94, and 83.58 tC/ha for VS1, VS2, VS3, VS4, and VS5, respectively (Figure 3 g). There was a statistically significant difference in organic soil carbon density between the forest types (χ^2^ = 1.7333, *p* = 0.230), but pairwise comparisons showed no significant differences (Additional file 1: 2k). These results are consistent with those of other studies of soil carbon stocks in wetlands (e.g. organic soil carbon stocks in swamp forests in Indonesia (with *Melaleuca* vegetation) were 106.00 and 135.63 tC/ha in the top 30 cm of soil of primary and secondary forests, respectively [29]).

Overall, the carbon density of *Melaleuca* forests on sandy soil in Southern Vietnam ranged from 159.36 tC/ha for regenerating forests to 275.98 tC/ha for primary forests. The carbon densities of forests on clay soil ranged from 246.96 tC/ha for regenerating forests without peat to 784.68 tC/ha of secondary forests with peat. Compared with the carbon stocks of other forests on peatland (e.g. the carbon density of mangrove forests in the Indo-Pacific region was 1,030 tC/ha [30]), the carbon density of disturbed *Melaleuca* forests on the peatland of Southern Vietnam is about one half, but the results are consistent with other studies on peat swamp forests (e.g. the carbon density of undisturbed swamp forests in South-East Asia ranged from 182 to 306 tC/ha [31]). Despite this, *Melaleuca* forests in the peatlands of Vietnam still have high potential as carbon stores. The case of U Minh Thuong National Park is an example. The total carbon stock of 8,038 ha of *Melaleuca* forests in the park is about 2.69 M tC (Table 2), which is equivalent 9.43 M tCO_2_e. Furthermore, there were 8,576 hectares of *Melaleuca* forested peatland in U Minh Ha National Park that have peat layers ranging from 40 cm to over 120 cm deep [32], which provides an even higher potential carbon store.

**Table 2:** Potential carbon storage in *Melaleuca* peat-swamp forests: case in U Minh Thuong National Park

Land cover type	Area (ha)	Carbon density (tC/ha)	Carbon storage tC
Mature *Melaleuca* forests on clay soil without peat	1,765	305.06	538,431
Mature *Melaleuca* forests on clay soil with peat	601	784.68	471,593
Regenerating *Melaleuca* on clay soil with peat	2,106	544.28	1,146,254
Regenerating *Melaleuca* on clay soil without peat	1,106	246.96	273,138
Others (open water, reeds and grasses)	2,460	107.91	265,459
Total	8,038		2,694,874

The areas of *Melaleuca* forests in U Minh Thuong National Park are taken from a Vietnam Environment Protection Agency report [48].

### Sodicity tolerance of *Melaleuca cajuputi* forests toward the adaptation to global climate change

Sea-level rise is a consequence of global climate change that will severely affect coastal and wetland ecosystems. *Melaleuca* forests are largely located in coastal and wetland areas that may be affected by climate change [33], so the risk of salinization of the region will increase. Salinity in soils can damage woody plant species by stunting buds, reducing leaf size and causing necroses in buds, roots, leaf margins and shoot tips [34]. Salinity can also inhibit seed germination, and can even kill non-halophytic species [35]. Both vegetative and reproductive growth of woody species are also reduced by high concentrations of sodium chloride in soil [35, 36]. The combination of flooding and salinity can create a more pronounced effect on growth and survival of plants than either stress alone [35]. High concentrations of sodium can affect the structure of sodic soils [37–39]. In contrast, low sodium concentration, soil structure is not affected by salinity in saline soil [40]. Sodicity and salinity always occur together and coming to have negative impacts on soil properties and plants [38, 41], but sodic soils may be either non-saline or saline [17].

The lower Mekong Basin and coastal regions of southern Vietnam are highly vulnerable to global climate change impacts [9, 33, 42, 43]. Most of Vietnam’s *Melaleuca* forests occur in these areas and will be affected projected sea-level rise. Fortunately, this study has shown that *M. cajuputi* has the ability to tolerant increase in sodic soils.

About 28 soil samples collected from *Melaleuca* forests in Southern Vietnam were examined and all were shown to be sodic (Table 3). While the exchangeable sodium percentage (ESP) of soil layers of *Melaleuca* forests on clay soil (VS3, VS4, and VS5) ranges from low to moderate sodicity, those of *Melaleuca* forests on sandy soil (VS1 and VS2) were significantly higher, particularly VS1, which had an ESP of up to 39.78% in soil taken from depths of 10–30 cm (Table 3). This indicates that both mature and young *M. cajuputi* forests have a high tolerance of sodic soils. Furthermore, *M. cajuputi* seeds can germinate and grow in highly sodic soil [e.g. *M. cajuputi* in forest type VS2 was able to grow in highly sodic soil with ESP up to 21.16% in the top 0–10 cm (Table 3)].

**Table 3:** Chemical element concentration and sodicity levels of the *Melaleuca* forest soils in the study areas

Forest types	Soil layers (cm)	pH_(KCl)_		Ca^2+^ (meq/100 g)		Mg^2+^ (meq/100 g)		Na^+^ (meq/100 g)		K^+^ (meq/100 g)		Al^3+^ (meq/100 g)		Fe^3+^ (mg/100 g)		ESP (%)		Sodicity
		Mean	SE	Mean	SE	Mean	SE	Mean	SE	Mean	SE	Mean	SE	Mean	SE	Mean	SE	
Primary *Melaleuca* on sandy soil (VS1)	0–10	3.97	0.15	1.413	0.75	1.783	1.58	1.790	1.56	0.600	0.47	0.910	0.29	3.303	1.20	32.05	4.28	High
	10–30	4.12	0.17	1.065	0.41	1.138	1.02	1.708	1.58	0.383	0.33	0.660	0.24	7.310	2.23	39.78	7.90	High
Regenerating *Melaleuca* on sandy soil (VS2)	0–10	3.68	0.03	0.690	0.10	0.310	0.10	0.310	0.15	0.155	0.02	1.860	0.14	1.615	0.36	21.16	7.82	High
	10–30	3.86	0.04	0.645	0.03	0.175	0.00	0.150	0.02	0.065	0.02	1.280	0.24	1.810	0.56	14.49	2.28	Moderate
Degraded secondary *Melaleuca* on clay soil with peat (VS3)	0–10	4.12	0.25	7.585	1.82	6.320	1.81	1.705	0.81	0.455	0.19	0.100	0.10	37.155	17.63	10.61	2.37	Moderate
	10–30	4.07	0.32	7.585	2.96	5.795	2.59	1.470	0.41	0.705	0.07	1.680	1.64	48.245	7.19	9.45	1.09	Low
Regenerating *Melaleuca* on clay soil with peat (VS4)	0–10	4.67	0.19	8.845	0.55	6.685	0.58	1.760	0.51	0.585	0.27	0.00	0.00	47.550	16.06	9.85	3.05	Low
	10–30	5.00	0.04	5.855	0.33	4.860	0.42	1.320	0.19	0.575	0.18	0.00	0.00	54.825	36.49	10.47	1.27	Moderate
Regenerating *Melaleuca* on clay soil without peat (VS5)	0–10	4.16	0.26	11.580	4.19	5.557	0.55	1.330	0.24	0.663	0.07	5.533	5.53	67.433	9.03	6.95	0.49	Low
	10–30	3.91	0.40	8.603	1.65	5.170	0.08	1.507	0.24	0.717	0.09	8.000	7.02	78.440	10.37	9.42	0.37	Low

With the exception of mangroves, few woody species can tolerate saline and/or sodic soils. Many woody species have been examined for their tolerance of salinity and/or sodicity. For example, *Eucalyptus*, *Melaleuca*, *Acacia*, *Casuarina* [21–24], *Grevillea robusta, Lophostemon confertus* and *Pinus caribea* [44], and *Moringa olifera* [45] have been examined and their tolerance to salinity assessed in the field and in glasshouses. In extremely saline soils in Australia, Niknam and McComb [22] suggested that the land care benefit of establishing species such as *Melaleuca* or *Casuarina* is more important than their commercial value. As well as the land care value, this study has shown that *M. cajuputi* forests in Vietnam can adapt to climate change through their tolerance to sodicity, and other harsh conditions [33], and can help to mitigate climate change through their carbon storage abilities.

## Conclusion

By undertaking original field data, this study examined the carbon sequestration potential of five types of *Melaleuca* forests including ‘Primary *Melaleuca* forests on sandy soil’ (VS1), ‘Regenerating *Melaleuca* forests on sandy soil’ (VS2), ‘Degraded secondary *Melaleuca* forests on clay soil with peat’ (VS3), ‘Regenerating *Melaleuca* forests on clay soil with peat’ (VS4), and ‘Regenerating *Melaleuca* forests on clay soil without peat’ (VS5). The study also assessed the sodicity tolerance of *M. cajuputi* forests in coastal and wetland regions of Vietnam.

The carbon densities of VS1, VS2, VS3, VS4, and VS5 were 275.98 (±38.62) tC/ha, 159.36 (±21.01) tC/ha, 784.68 (±54.72) tC/ha, 544.28 (±56.26) tC/ha, and 246.96 (±27.56) tC/ha, respectively. Most carbon stocks were contributed from the soil (including peat) and stands.

The exchangeable sodium percentage (ESP) of soil from *Melaleuca* forests on clay soil (VS3, VS4, and VS5) ranged from low to moderate sodicity, but those from *Melaleuca* forests on sandy soil (VS1 and VS2) were highly sodic.

The results provide important information for the future sustainable management of *Melaleuca* forests in Vietnam, particularly in regards to forest carbon conservation initiatives and the potential of *Melaleuca* species for reforestation initiatives on degraded sites with highly sodic soils. In Vietnam, forest carbon conservation initiatives such as REDD+ have hereto, in our view, not placed appropriate priority or consideration on the protection of carbon stocks of *Melaleuca* forests. The results presented in this paper suggest that *Melaleuca* forests in Vietnam, particularly those on peatland areas, hold globally significant carbon stocks—arguably greater than those found in upland rainforest ecosystems, which have so far been given higher priority in REDD+ planning in Vietnam. Furthermore, the results presented in this paper suggest that some *Melaleuca* forest species in Vietnam, particularly those on sandy soils, exhibit a tolerance for highly sodic soils. This suggests that those species might be useful in reforestation initiatives on degraded sites with highly sodic soils. As degradation pressures including climate change continue to alter the hydrological features of soil systems in areas such as the Mekong Delta in Vietnam, and the sodicity of soils in some areas increases, *Melaleuca* species could offer a useful option for reforestation and rehabilitation initiatives.

The results in this research provide further scientific information to support better *Melaleuca* ecosystem management. The results should help policy makers make better decisions in an era of global change. The results have particular relevance for the application of REED+ in the Southeast Asia.

## Methods

### Study sites and disturbance context

**Figure 4:**
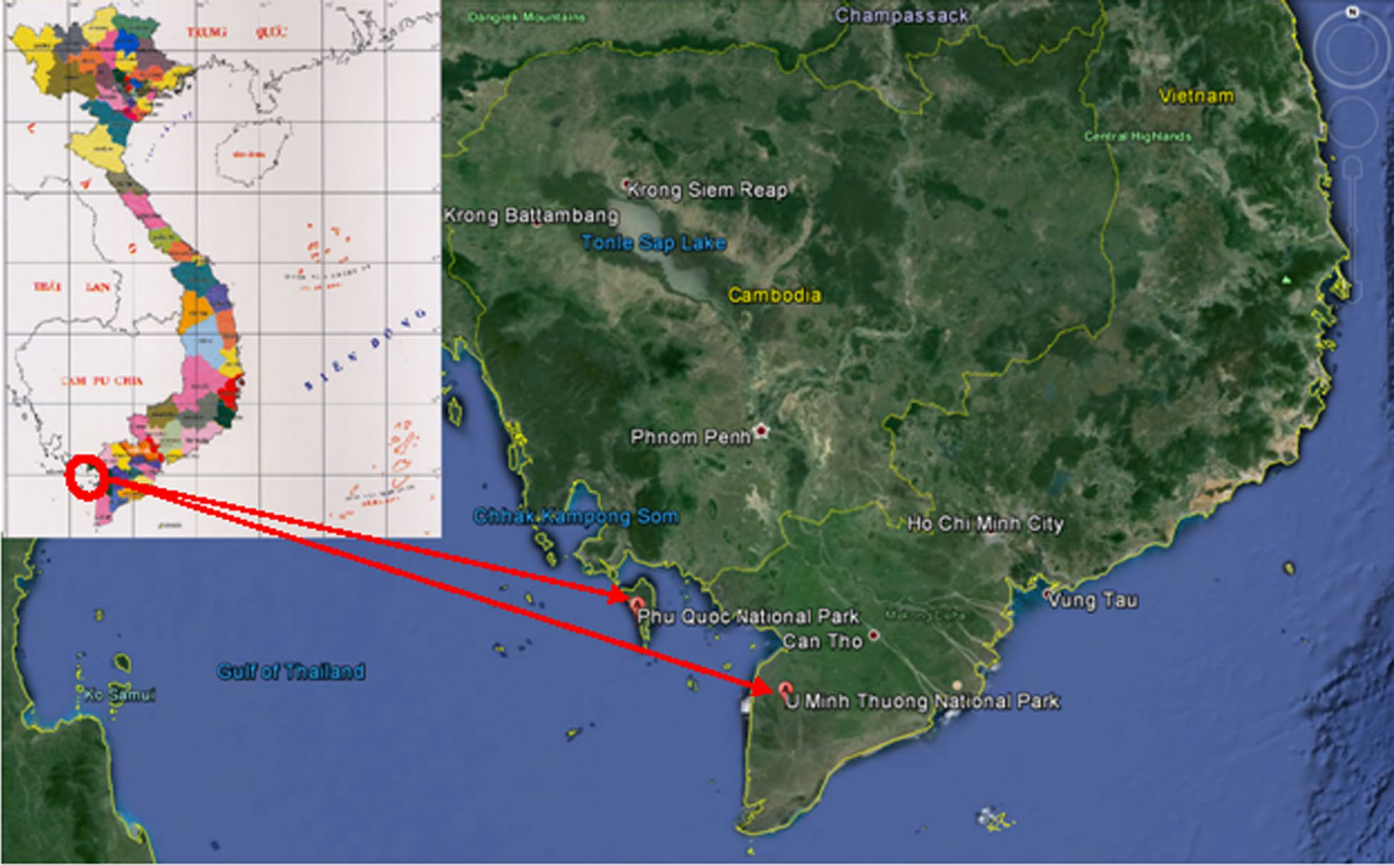
The study locations in Southern Vietnam: Phu Quoc National Park and U Minh Thuong National Park. Source: map from Department of Information Technology, Vietnam. Image Landsat from Google Earth (free version).

*Melaleuca cajuputi* is naturally distributed as scattered shrub populations along the coastal regions in the middle Provinces and up to the Northern hilly regions, and as tall forests in the Mekong Delta of Vietnam [46]. Thus, the study focussed on the sites in Southern Vietnam (involving Mekong Delta). The study investigated two sites: the Phu Quoc National Park and U Minh Thuong National Park, which both contain extensive *Melaleuca* forests in coastal wetlands (Figure 4). A total of 14 plots were randomly selected for carbon storage assessment, covering five types of *Melaleuca* stands: ‘Primary *Melaleuca* forests on sandy soil’(VS1), 4 plots; ‘Regenerating *Melaleuca* forests on sandy soil’ (VS2), 2 plots; ‘Degraded secondary *Melaleuca* forests on clay soil with peat’ (VS3), 2 plots; ‘Regenerating *Melaleuca* forests on clay soil with peat’ (VS4), 3 plots; and ‘Regenerating *Melaleuca* forests on clay soil without peat’ (VS5), 3 plots.

Phu Quoc National Park is located on the northern Phu Quoc Island of Vietnam (at N 10°12′07″–N 10°27′02″, E 103°50′04″–E 104°04′40″) (Figure 4). *Melaleuca* forest areas cover 1,667.50 ha out of the total area of 28,496.90 ha. These *Melaleuca* forests naturally occur on lowland regions of the island where they are seasonally inundated and/or permanent saturated, and also on permanent sand bars where no inundation occurs [47]. The rest areas of the park are hilly and mountainous forests. Two *Melaleuca* forest types were found in the park: primary *Melaleuca* forest (VS1); and regenerating *Melaleuca* forest (VS2). Before the park was established in 2001, key disturbance included forest fires and human intrusion for crop cultivation. The regenerating *Melaleuca* forests were up to 10–12 years of age at the time this study was conducted.

U Minh Thuong National Park is located in the Kien Giang Province (at N 9° 31′–N 9° 39′, E 105° 03′–E 105° 07′) (Figure 4). *Melaleuca* forest on swamp peatland is an endemic ecosystem in the lower Mekong Basin of Vietnam. The core area of the park is 8,038 ha, which is surrounded by a buffer zone of 13,069 ha. Here, the key disturbance is fire, with the last major fire occurring in April 2002, which burnt the primary vegetation as well as the peat soil. The Vietnamese Environment Protection Agency [48] reported that 3,212 hectares of *Melaleuca* forests was almost destroyed, so a canal system was built as a key management solution to increase water inundation of the forest to prevent fires. Currently, there are three *Melaleuca* forest types in U Minh Thuong National Park: VS3, VS4, and VS5. At the time of this study, the VS4 and VS5 areas were up to 10 years old.

### Field sampling and data collection

The major plots were set out as 500 m^2^ quadrats (20 m × 25 m), and all trees with a DBH ≥ 10 cm were measured and recorded. Sub-plots also were set out as 100 m^2^ quadrats (20 m × 5 m) within the major plots to measure all trees with DBH < 10 cm and a total height of >1.3 m (modified from Van et al. [49]). Data on DBH, alive or dead, and height were recorded for all standing trees.

Deadwood (dead fallen trees) with a diameter ≥10 cm were measured within the major plots (500 m^2^), while deadwood with 5 cm ≤ diameter < 10 cm were measured within the sub-plots (100 m^2^). Diameters at both ends of the trunk (D1 and D2), length (if ≥50 cm length), and the decay classes (involved sound, intermediate, and rotten [50, 51]) were recorded for all deadwood.

Seventy random quadrats (1 m × 1 m) were located in the main plots to collect and record the ‘fresh weight’ of the understory. Samples of all species from the understory were collected in each major plot and taken back to the Vietnam Forestry University laboratory for drying.

Seventy random coarse litter samples and seventy random fine litter samples were collected in the major plots. The fresh weight of each litter sample was recorded. Each litter type (coarse litter and fine litter) collected in every major plot were well mixed and taken to the laboratory for drying.

Two soil samples, one from the upper (0–10 cm) soil layer and one from the lower (10–30 cm) soil layer, were taken from each of 14 plots, giving a total of 28 soil samples. The 28 soil samples were taken back to the National Institute of Agricultural Planning and Projection laboratory for further analysis. Various soil chemical properties of the 28 samples were tested including: pH_KCl_, total C, total N, Ca^2+^, Mg^2+^, Na^+^, K^+^, Al^3+^, and Fe^3+^. Twenty-eight duplicate soil samples were collected and analyzed for bulk density.

### Sample analysis

Each understory and litter sample was divided into three sub-samples and dried in a drying oven at 60°C to measure the moisture content, based on the Eq. (1) below:

1 $$R_{moist} = \frac{{\mathop \sum \nolimits_{i = 1}^{n} \frac{{W_{fi} - W_{di} }}{{W_{fi} }}}}{n}.$$

where *R*_*moist*_ = moist ratio [0:1], *W*_*fi*_ = fresh weight of sub-sample i, *W*_*di*_ = dry weight of sub-sample i, n = number of sub-samples. The scales used to weight sub-samples were accurate to ±0.01 g.

Total organic carbon (C%) was measured using the Walkley–Black method, which is commonly used to examine soil organic carbon via oxidation with K_2_Cr_2_O_7_ [52, 53]. Total nitrogen was measured using the Kjeldahl method, which is the standard way to determine the total organic nitrogen content of soil [54]. A standard bulk density test was used to analyze all soil bulk samples in a dryven. Bulk density was calculated using Eq. (2):

2 $$BD = \frac{Ms}{V}.$$

where *BD* = the bulk density of the oven-dry soil sample (g/cm^3^), *Ms* = the oven dry-mass of the soil sample (gram), *V* = the volume of the ring sample (cm^3^).

Exchangeable sodium percentage (*ESP*) was calculated using Eq. (3) [55–57], and classified with four sodic levels as non-sodic soil (*ESP* < 6), low sodic soil (*ESP* = 6–10), moderately sodic soil (*ESP* = 10–15), and highly sodic soil (*ESP* > 15) [55–57].

3 $$ESP = \frac{{{\text{Na}}^{ + } }}{{\varSigma \left[ {{\text{Na}}^{ + } } \right]\left[ {{\text{K}}^{ + } } \right]\left[ {{\text{Mg}}^{2 + } } \right]\left[ {{\text{Ca}}^{2 + } } \right]}} \times 100.$$

Basal area (*BA*) was calculated with Eq. (4) (modified from Jonson and Freudenberger [58]):

4 $$BA = \frac{{\mathop \sum \nolimits_{n}^{1} \left[ {\pi \times ( DBH_{i}/200)^{2} } \right]}}{{S_{plot} }} \times 10,000$$

where *BA* = basal area (m^2^/ha), *DBH*_*i*_ = diameter at bread height of tree i (cm), *i* = stand individual (i = [1:n]), *n* = number of trees of sample plot, *S*_*plot*_ = area of the sample plot (m^2^).

### Biomass allometric computation

Nine allometric equations, which are most common way to measure forest carbon stocks, were applied to calculate the above-ground and root biomass of the stands (Table 4). The selected allometric equations were tested for statistical significance using the R Statistic Program (Additional file 1: 1). Using these equations, the average biomass was analyzed for five typical *Melaleuca* stands (VS1, VS2, VS3, VS4, and VS5). To convert from fresh to dry biomass, a moisture rate of 0.5 was applied as suggested by Van et al. [49] for the allometric equation of Finlayson et al. [59]. According to the Global Wood Density Database, the density of *M. cajuputi* timber ranges from 0.6 to 0.87 g/cm^3^ [60], so 0.6 g/cm^3^ was applied for the above-ground biomass allometric equation of Chave et al. [61].

**Table 4:** List of allometric equations applied to examine stand biomass of the *Melaleuca* forests

Allometric equations	R^2^	Vegetation	Sites	References
log_10_(*FW*) = 2.266log_10_(*D*) − 0.502 where *FW* = fresh above-ground biomass (kg/tree), *D* = diameter at breast height (cm)	0.98	*Melaleuca* spp.	Northern Territory	Finlayson et al. [59]
*y* = 0.124 × *DBH* ^2.247^ where *y* = above-ground biomass (kg/tree), *DBH* = diameter at breast height (cm)	0.97	*Melaleuca cajuputi*	Vietnam	Le [63]
*y* = exp[–2.134 + 2.53ln(*D*)] where *y* = above-ground biomass (kg/tree), *D* = diameter at breast height (cm)	0.97	Mixed species	Tropical, moist forest	IPCC [51] or Brown [64]
ln(*y*) = 2.4855ln(*x*) − 2.3267 where *y* = above-ground biomass (kg/tree), *x* = diameter at breast height (cm)	0.96	Native sclerophyll forest	NSW, ACT, VIC, TAS, and SA	Keith et al. [65]
ln(*AGB*) = –1,554 + 2.420ln(*D*) + ln(*ρ*) where *AGB* = above-ground biomass (kg/tree), *D* = diameter at breast height (cm), *ρ* = wood density (g/cm^3^)	0.99	Tropical forests	America, Asian and Oceania	Chave et al. [61]
ln(*RBD*) = – 1,085 + 0.926ln(ABD) where *RBD* = root biomass density (tons/ha), *ABD* = above-ground biomass density (tons/ha)	0.83	Upland forests	Worldwide	IPCC [51] or Cairn et al. [66]
*y* = 0.27*x* where *y* = total root biomass (tons/ha), *x* = total shoot biomass (tons/ha)	0.81	Natural forests	Worldwide	Mokany et al. [67]
*Wr* = 0.0214 × *D* ^2.33^ where *W*_*r*_ = coarse root biomass (kg/tree), *D* = diameter at breast height (cm)	0.94	Tropical secondary forests	Sarawak, Malaysia	Kenzo et al. [68]
*W*_*r*_ = 0.023 × *D* ^2.59^ where W_r_ = coarse root biomass (kg/tree), *D* = diameter at breast height (cm)	0.97	Tropical secondary forests	Sarawak, Malaysia	Niiyama et al. [69]

*NSW* New South Wales, *ACT* Australian Capital Territory, *VIC* Victoria, *TAS* Tasmania, *SA* South Australia.

The fallen deadwood biomass were calculated using Eq. (5) ([62], p 12):

5 $$B = \pi \times r^{2} \times L \times \delta$$

where *B* = biomass (kg), *r* = ½ diameter (cm), *L* = length (m), and *δ* = wood density (= 0.6 g/cm^3^).

Then, the biomass of the fallen deadwood was determined using the IPCC [50, 51] density reduction factors (sound = 1, intermediate = 0.6, and rotten = 0.45). The biomass of standing dead trees was measured using the same criteria as live trees, but a reduction factor of 0.975 is applied to dead trees that have lost leaves and twigs, and 0.8 for dead trees that have lost leaves, twigs, and small branches (diameter <10 cm) ([51], p 4.105).

To convert biomass to carbon mass for all categories (stands, roots, deadwood, understory, and litter), a factor of 0.45 was applied.

Soil organic carbon (SOC) was calculated using Eq. (6) [50, 51]:

6 $$SOC = Dep \times BD \times C_{sample} \times 100$$

where *SOC* = Soil organic carbon, *Dep* = depth of soil layer (m), *BD* = bulk density (g/cm^3^), *C*_*sample*_ = organic carbon content of soil sample (%), and 100 is the default unit conversion factor.

### Statistical analysis

One-way ANOVA tests were applied to compare stand densities, DBH, height classes, basal areas, and six categories of carbon stocks of the five *Melaleuca* forest types. LSD post hoc tests were also used for all pairwise comparisons between group means. Statistical analysis was undertaken using Microsoft Excel 2010 and the R Statistic Program.

## Additional file

**Additional file 1:** Data analysis.
