# Supplementary material for: An assessment of the carbon stocks and sodicity tolerance of disturbed Melaleuca forests in Southern Vietnam
Source: Carbon Balance Manag. 2015 Jul 14;10:15. doi: 10.1186/s13021-015-0025-6 (PMC4500848; doi:10.1186/s13021-015-0025-6)
Supplement: Supplementary file 1 — Additional file 1: Data analysis. [file 13021_2015_25_MOESM1_ESM.docx]

**An assessment of the carbon stocks and sodicity tolerance of disturbed *Melaleuca* forests in Southern Vietnam**

Da B. Tran^1^*, Tho V. Hoang^1^, Paul Dargusch^2^

^1^ Vietnam Forestry University, Hanoi, VIETNAM

^2^ School of Geography, Planning and Environmental Management, the University of Queensland Australia, Brisbane QLD, AUSTRALIA

*Correspondence author: [tranbinhda@gmail.com](mailto:tranbinhda@gmail.com)

**Supplementary 1: ALLOWMETRIC EQUATION TESTS**

**Supplementary 1a: Summary of pairwise comparisons of allometric equation tests for above-ground biomass**

| Comparisons | AGB.Ray | AGB.Fin | AGB.Keith | AGB.Le | AGB.IPCC | AGB.Chave |
| --- | --- | --- | --- | --- | --- | --- |
| AGB.Ray |  | F, F, F, **T**, F, **T,** **T**, **T**, **T**, **T**, **T**, **T**, **T**, F | **T**, F, F, **T**, F, F, **T**, **T**, **T**, **T**, **T**, **T**, **T**, F | F, F, F, **T**, F, **T**, **T**, **T**, **T**, **T**, **T**, **T**, **T**, F | F, F, F, **T**, F, **T**, **T**, **T**, **T**, **T**, **T**, **T**, **T**, **T** | F, F, F, **T**, F, **T**, **T**, **T**, **T**, **T**, **T**, **T**, **T**, F |
| AGB.Fin |  |  | F, F, F, F, F, F, F, F, F, F, F, F, F, F | F, F, F, F, F, F, F, F, F, F, F, F, F, F | F, F, F, F, F, F, F, F, **T,** F, F, F, F, F | F, F, F, F, F, F, F, F, F, F, F, F, F, F |
| AGB.Keith |  |  |  | F, F, F, F, F, F, F, F, F, F, F, F, F, F | F, F, F, F, F, F, F, F, F, F, F, F, F, F | F, F, F, F, F, F, F, F, F, F, F, F, F, F |
| AGB.Le |  |  |  |  | F, F, F, F, F, F, F, F, **T**, F, F, F, F, F | F, F, F, F, F, F, F, F, F, F, F, F, F, F |
| AGB.IPCC |  |  |  |  |  | F, F, F, F, F, F, F, F, F, F, F, F, F, F |
| AGB.Chave |  |  |  |  |  |  |

**Supplementary 1b: Percentages of pairwise comparisons of allometric equation tests for above-ground biomass**

| Stand biomass equations | References | Codes | Count “T” | % | Count “F” | % |
| --- | --- | --- | --- | --- | --- | --- |
| ln(y) = 2.0409ln(D) – 2.0163 | Rayachhetry *et al.* ([2001](#_ENREF_83)) | AGB.Ray | 46 | 65.71 | 24 | 34.29 |
| Log_10_(FW) = 2.266log_10_(D) – 0.502 | Finlayson *et al.* ([1993](#_ENREF_10)) | AGB.Fin | 10 | 14.29 | 60 | 85.71 |
| ln(y) = 2.4855ln(x) – 2.3267] | Keith *et al.* ([2000](#_ENREF_16)) | AGB.Keith | 9 | 12.86 | 61 | 87.14 |
| y = 0.124 ˟ DBH 2.247 | Le (2005) | AGB.Le | 10 | 14.29 | 60 | 85.71 |
| y = exp[–2.134 + 2.53ln(D)] | IPCC ([2003](#_ENREF_13)) or Brown ([1997](#_ENREF_2)) | AGB.IPCC | 12 | 17.14 | 58 | 82.86 |
| ln(AGB) = – 1,554 + 2.420ln(D) +ln(r) | Chave *et al.* ([2005](#_ENREF_6)) | AGB.Chave | 9 | 12.86 | 61 | 87.14 |

> testEquVN <- read.csv("K:/PhD PROGRAM/A-PhD PROPOSAL/FIELD DATA/DATA ANALYSIS/CSV FILES/VN/testEquVN.csv")

| **Plot PQ1**  > qqnorm(pq1)  > qqline(pq1)  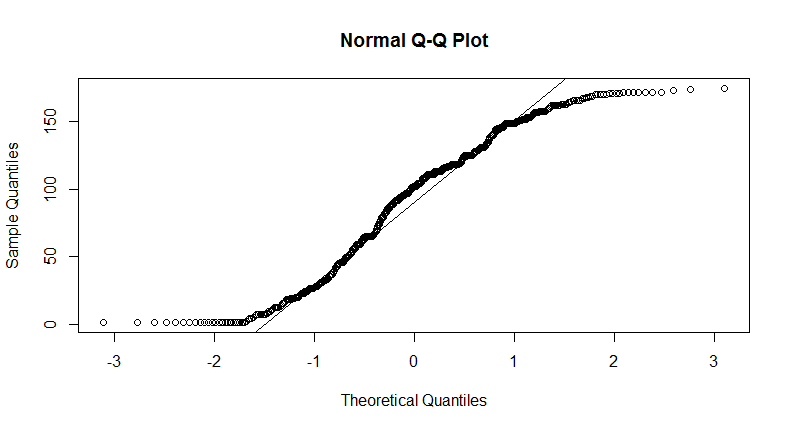 | > kruskal.test(pq1~eq1)  Kruskal-Wallis rank sum test  data: pq1 by eq1  Kruskal-Wallis chi-squared = 81.5227, df = 6, p-value = 1.731e-15  > kruskalmc(pq1, eq1)  Multiple comparison test after Kruskal-Wallis  p.value: 0.05  Comparisons  obs.dif critical.dif difference  AGB.Chave - AGB.Fin 11.095238 71.52007 FALSE  AGB.Chave - AGB.IPCC 25.190476 71.52007 FALSE  AGB.Chave - AGB.Keith 20.083333 71.52007 FALSE  AGB.Chave - AGB.Le 4.988095 71.52007 FALSE  AGB.Chave - AGB.Ray 51.619048 71.52007 FALSE  AGB.Fin - AGB.IPCC 36.285714 71.52007 FALSE  AGB.Fin - AGB.Keith 8.988095 71.52007 FALSE  AGB.Fin - AGB.Le 6.107143 71.52007 FALSE  AGB.Fin - AGB.Ray 62.714286 71.52007 FALSE  AGB.IPCC - AGB.Keith 45.273810 71.52007 FALSE  AGB.IPCC - AGB.Le 30.178571 71.52007 FALSE  AGB.IPCC - AGB.Ray 26.428571 71.52007 FALSE  AGB.Keith - AGB.Le 15.095238 71.52007 FALSE  AGB.Keith - AGB.Ray 71.702381 71.52007 TRUE  AGB.Le - AGB.Ray 56.607143 71.52007 FALSE |
| --- | --- |

| **Plot PQ2**  > qqnorm(pq2)  > qqline(pq2)  > 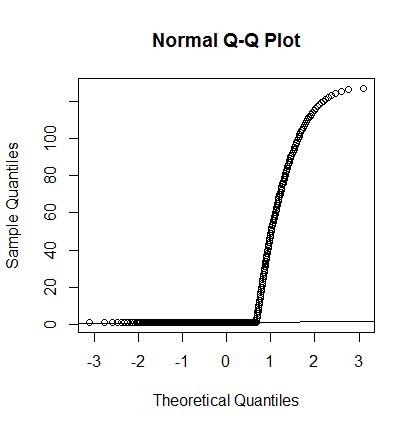 | > kruskal.test(pq2~eq2)  Kruskal-Wallis rank sum test  data: pq2 by eq2  Kruskal-Wallis chi-squared = 513.2837, df = 6, p-value < 2.2e-16  > kruskalmc(pq2, eq2)  Multiple comparison test after Kruskal-Wallis  p.value: 0.05  Comparisons  obs.dif critical.dif difference  AGB.Chave - AGB.Fin 12.318182 139.7514 FALSE  AGB.Chave - AGB.IPCC 4.954545 139.7514 FALSE  AGB.Chave - AGB.Keith 2.090909 139.7514 FALSE  AGB.Chave - AGB.Le 8.454545 139.7514 FALSE  AGB.Chave - AGB.Ray 14.090909 139.7514 FALSE  AGB.Fin - AGB.IPCC 17.272727 139.7514 FALSE  AGB.Fin - AGB.Keith 14.409091 139.7514 FALSE  AGB.Fin - AGB.Le 3.863636 139.7514 FALSE  AGB.Fin - AGB.Ray 1.772727 139.7514 FALSE  AGB.IPCC - AGB.Keith 2.863636 139.7514 FALSE  AGB.IPCC - AGB.Le 13.409091 139.7514 FALSE  AGB.IPCC - AGB.Ray 19.045455 139.7514 FALSE  AGB.Keith - AGB.Le 10.545455 139.7514 FALSE  AGB.Keith - AGB.Ray 16.181818 139.7514 FALSE  AGB.Le - AGB.Ray 5.636364 139.7514 FALSE |
| --- | --- |

| **Plot PQ3**  > qqnorm(pq3)  > qqline(pq3)  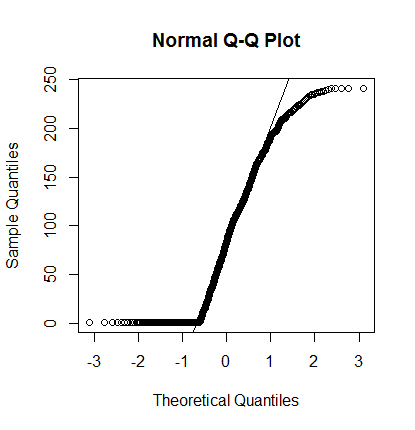 | > kruskal.test(pq3~eq3)  Kruskal-Wallis rank sum test  data: pq3 by eq3  Kruskal-Wallis chi-squared = 321.4749, df = 6, p-value < 2.2e-16  > kruskalmc(pq3, eq3)  Multiple comparison test after Kruskal-Wallis  p.value: 0.05  Comparisons  obs.dif critical.dif difference  AGB.Chave - AGB.Fin 8.812500 81.93653 FALSE  AGB.Chave - AGB.IPCC 22.984375 81.93653 FALSE  AGB.Chave - AGB.Keith 9.953125 81.93653 FALSE  AGB.Chave - AGB.Le 0.656250 81.93653 FALSE  AGB.Chave - AGB.Ray 4.906250 81.93653 FALSE  AGB.Fin - AGB.IPCC 14.171875 81.93653 FALSE  AGB.Fin - AGB.Keith 1.140625 81.93653 FALSE  AGB.Fin - AGB.Le 8.156250 81.93653 FALSE  AGB.Fin - AGB.Ray 13.718750 81.93653 FALSE  AGB.IPCC - AGB.Keith 13.031250 81.93653 FALSE  AGB.IPCC - AGB.Le 22.328125 81.93653 FALSE  AGB.IPCC - AGB.Ray 27.890625 81.93653 FALSE  AGB.Keith - AGB.Le 9.296875 81.93653 FALSE  AGB.Keith - AGB.Ray 14.859375 81.93653 FALSE  AGB.Le - AGB.Ray 5.562500 81.93653 FALSE |
| --- | --- |

| **Plot PQ4**  > qqnorm(pq4)  > qqline(pq4)  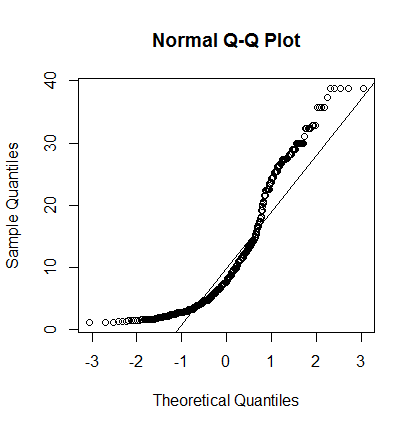 | > kruskal.test(pq4~eq4)  Kruskal-Wallis rank sum test  data: pq4 by eq4  Kruskal-Wallis chi-squared = 27.6855, df = 5, p-value = 4.193e-05  > kruskalmc(pq4, eq4)  Multiple comparison test after Kruskal-Wallis  p.value: 0.05  Comparisons  obs.dif critical.dif difference  AGB.Chave - AGB.Fin 9.8310811 64.08825 FALSE  AGB.Chave - AGB.IPCC 17.2364865 64.08825 FALSE  AGB.Chave - AGB.Keith 20.9391892 64.08825 FALSE  AGB.Chave - AGB.Le 20.0743243 64.08825 FALSE  AGB.Chave - AGB.Ray 85.3378378 64.08825 TRUE  AGB.Fin - AGB.IPCC 27.0675676 64.08825 FALSE  AGB.Fin - AGB.Keith 11.1081081 64.08825 FALSE  AGB.Fin - AGB.Le 10.2432432 64.08825 FALSE  AGB.Fin - AGB.Ray 75.5067568 64.08825 TRUE  AGB.IPCC - AGB.Keith 38.1756757 64.08825 FALSE  AGB.IPCC - AGB.Le 37.3108108 64.08825 FALSE  AGB.IPCC - AGB.Ray 102.5743243 64.08825 TRUE  AGB.Keith - AGB.Le 0.8648649 64.08825 FALSE  AGB.Keith - AGB.Ray 64.3986486 64.08825 TRUE  AGB.Le - AGB.Ray 65.2635135 64.08825 TRUE |
| --- | --- |

| **Plot PQ5**  > qqnorm(pq5)  > qqline(pq5)  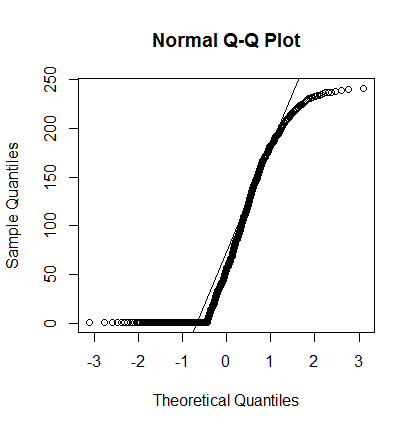 | > kruskal.test(pq5~eq5)  Kruskal-Wallis rank sum test  data: pq5 by eq5  Kruskal-Wallis chi-squared = 364.3507, df = 6, p-value < 2.2e-16  > kruskalmc(pq5, eq5)  Multiple comparison test after Kruskal-Wallis  p.value: 0.05  Comparisons  obs.dif critical.dif difference  AGB.Chave - AGB.Fin 5.169492 85.33782 FALSE  AGB.Chave - AGB.IPCC 14.389831 85.33782 FALSE  AGB.Chave - AGB.Keith 6.779661 85.33782 FALSE  AGB.Chave - AGB.Le 2.966102 85.33782 FALSE  AGB.Chave - AGB.Ray 34.203390 85.33782 FALSE  AGB.Fin - AGB.IPCC 9.220339 85.33782 FALSE  AGB.Fin - AGB.Keith 1.610169 85.33782 FALSE  AGB.Fin - AGB.Le 2.203390 85.33782 FALSE  AGB.Fin - AGB.Ray 29.033898 85.33782 FALSE  AGB.IPCC - AGB.Keith 7.610169 85.33782 FALSE  AGB.IPCC - AGB.Le 11.423729 85.33782 FALSE  AGB.IPCC - AGB.Ray 19.813559 85.33782 FALSE  AGB.Keith - AGB.Le 3.813559 85.33782 FALSE  AGB.Keith - AGB.Ray 27.423729 85.33782 FALSE  AGB.Le - AGB.Ray 31.237288 85.33782 FALSE |
| --- | --- |

| **Plot PQ6**  > qqnorm(pq6)  > qqline(pq6)  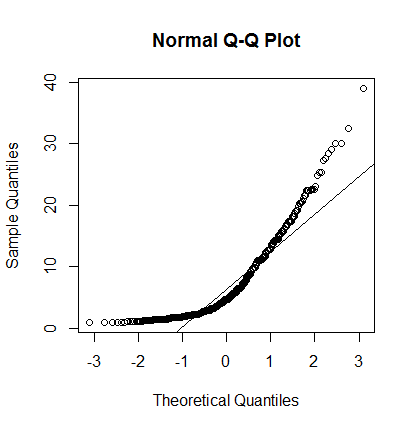 | > kruskal.test(pq6~eq6)  Kruskal-Wallis rank sum test  data: pq6 by eq6  Kruskal-Wallis chi-squared = 28.3659, df = 5, p-value = 3.087e-05  > kruskalmc(pq6, eq6)  Multiple comparison test after Kruskal-Wallis  p.value: 0.05  Comparisons  obs.dif critical.dif difference  AGB.Chave - AGB.Fin 2.806818 67.50959 FALSE  AGB.Chave - AGB.IPCC 18.170455 67.50959 FALSE  AGB.Chave - AGB.Keith 26.272727 67.50959 FALSE  AGB.Chave - AGB.Le 18.681818 67.50959 FALSE  AGB.Chave - AGB.Ray 92.863636 67.50959 TRUE  AGB.Fin - AGB.IPCC 20.977273 67.50959 FALSE  AGB.Fin - AGB.Keith 23.465909 67.50959 FALSE  AGB.Fin - AGB.Le 15.875000 67.50959 FALSE  AGB.Fin - AGB.Ray 90.056818 67.50959 TRUE  AGB.IPCC - AGB.Keith 44.443182 67.50959 FALSE  AGB.IPCC - AGB.Le 36.852273 67.50959 FALSE  AGB.IPCC - AGB.Ray 111.034091 67.50959 TRUE  AGB.Keith - AGB.Le 7.590909 67.50959 FALSE  AGB.Keith - AGB.Ray 66.590909 67.50959 FALSE  AGB.Le - AGB.Ray 74.181818 67.50959 TRUE |
| --- | --- |

| **Plot UM1**  > qqnorm(um1)  > qqline(um1)  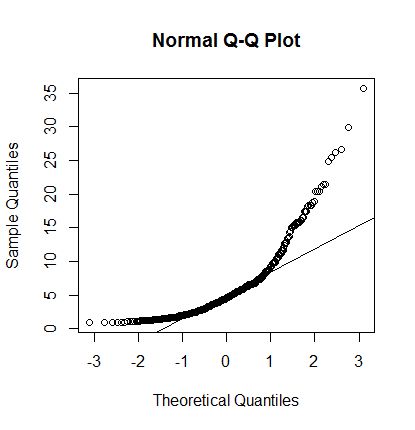 | > kruskal.test(um1~equm1)  Kruskal-Wallis rank sum test  data: um1 by equm1  Kruskal-Wallis chi-squared = 38.6443, df = 5, p-value = 2.8e-07  > kruskalmc(um1~equm1)  Multiple comparison test after Kruskal-Wallis  p.value: 0.05  Comparisons  obs.dif critical.dif difference  AGB.Chave - AGB.Fin 3.414773 67.50959 FALSE  AGB.Chave - AGB.IPCC 19.318182 67.50959 FALSE  AGB.Chave - AGB.Keith 32.937500 67.50959 FALSE  AGB.Chave - AGB.Le 24.136364 67.50959 FALSE  AGB.Chave - AGB.Ray 109.272727 67.50959 TRUE  AGB.Fin - AGB.IPCC 22.732955 67.50959 FALSE  AGB.Fin - AGB.Keith 29.522727 67.50959 FALSE  AGB.Fin - AGB.Le 20.721591 67.50959 FALSE  AGB.Fin - AGB.Ray 105.857955 67.50959 TRUE  AGB.IPCC - AGB.Keith 52.255682 67.50959 FALSE  AGB.IPCC - AGB.Le 43.454545 67.50959 FALSE  AGB.IPCC - AGB.Ray 128.590909 67.50959 TRUE  AGB.Keith - AGB.Le 8.801136 67.50959 FALSE  AGB.Keith - AGB.Ray 76.335227 67.50959 TRUE  AGB.Le - AGB.Ray 85.136364 67.50959 TRUE |
| --- | --- |

| **Plot UM2**  > qqnorm(um2)  > qqline(um2)  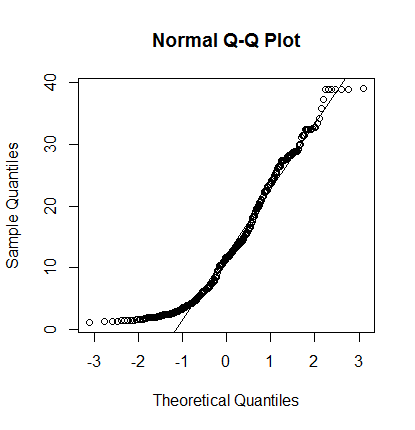 | > kruskal.test(um2~equm2)  Kruskal-Wallis rank sum test  data: um2 by equm2  Kruskal-Wallis chi-squared = 50.1692, df = 5, p-value = 1.28e-09  > kruskalmc(um1,equm1)  Multiple comparison test after Kruskal-Wallis  p.value: 0.05  Comparisons  obs.dif critical.dif difference  AGB.Chave - AGB.Fin 3.414773 67.50959 FALSE  AGB.Chave - AGB.IPCC 19.318182 67.50959 FALSE  AGB.Chave - AGB.Keith 32.937500 67.50959 FALSE  AGB.Chave - AGB.Le 24.136364 67.50959 FALSE  AGB.Chave - AGB.Ray 109.272727 67.50959 TRUE  AGB.Fin - AGB.IPCC 22.732955 67.50959 FALSE  AGB.Fin - AGB.Keith 29.522727 67.50959 FALSE  AGB.Fin - AGB.Le 20.721591 67.50959 FALSE  AGB.Fin - AGB.Ray 105.857955 67.50959 TRUE  AGB.IPCC - AGB.Keith 52.255682 67.50959 FALSE  AGB.IPCC - AGB.Le 43.454545 67.50959 FALSE  AGB.IPCC - AGB.Ray 128.590909 67.50959 TRUE  AGB.Keith - AGB.Le 8.801136 67.50959 FALSE  AGB.Keith - AGB.Ray 76.335227 67.50959 TRUE  AGB.Le - AGB.Ray 85.136364 67.50959 TRUE |
| --- | --- |

| **Plot UM3**  > qqnorm(um3)  > qqline(um3)  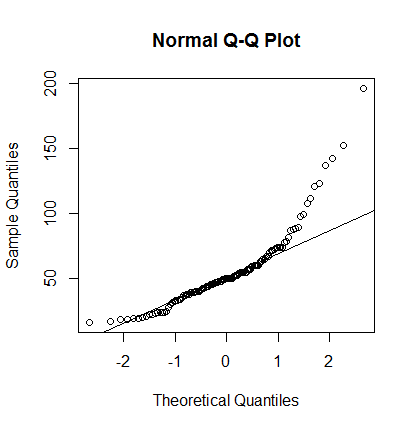 | > kruskal.test(um3~equm3)  Kruskal-Wallis rank sum test  data: um3 by equm3  Kruskal-Wallis chi-squared = 61.4215, df = 5, p-value = 6.178e-12  > kruskalmc(um3,equm3)  Multiple comparison test after Kruskal-Wallis  p.value: 0.05  Comparisons  obs.dif critical.dif difference  AGB.Chave - AGB.Fin 17.857143 34.23736 FALSE  AGB.Chave - AGB.IPCC 18.857143 34.23736 FALSE  AGB.Chave - AGB.Keith 9.333333 34.23736 FALSE  AGB.Chave - AGB.Le 19.714286 34.23736 FALSE  AGB.Chave - AGB.Ray 64.666667 34.23736 TRUE  AGB.Fin - AGB.IPCC 36.714286 34.23736 TRUE  AGB.Fin - AGB.Keith 8.523810 34.23736 FALSE  AGB.Fin - AGB.Le 1.857143 34.23736 FALSE  AGB.Fin - AGB.Ray 46.809524 34.23736 TRUE  AGB.IPCC - AGB.Keith 28.190476 34.23736 FALSE  AGB.IPCC - AGB.Le 38.571429 34.23736 TRUE  AGB.IPCC - AGB.Ray 83.523810 34.23736 TRUE  AGB.Keith - AGB.Le 10.380952 34.23736 FALSE  AGB.Keith - AGB.Ray 55.333333 34.23736 TRUE  AGB.Le - AGB.Ray 44.952381 34.23736 TRUE |
| --- | --- |

| **Plot UM4**  > qqnorm(um4)  > qqline(um4)  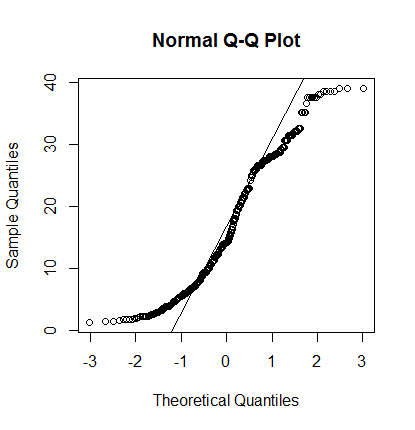 | > kruskal.test(um4~equm4)  Kruskal-Wallis rank sum test  data: um4 by equm4  Kruskal-Wallis chi-squared = 52.309, df = 5, p-value = 4.662e-10  > kruskalmc(um4, equm4)  Multiple comparison test after Kruskal-Wallis  p.value: 0.05  Comparisons  obs.dif critical.dif difference  AGB.Chave - AGB.Fin 21.666667 60.5332 FALSE  AGB.Chave - AGB.IPCC 24.356061 60.5332 FALSE  AGB.Chave - AGB.Keith 23.575758 60.5332 FALSE  AGB.Chave - AGB.Le 31.340909 60.5332 FALSE  AGB.Chave - AGB.Ray 110.409091 60.5332 TRUE  AGB.Fin - AGB.IPCC 46.022727 60.5332 FALSE  AGB.Fin - AGB.Keith 1.909091 60.5332 FALSE  AGB.Fin - AGB.Le 9.674242 60.5332 FALSE  AGB.Fin - AGB.Ray 88.742424 60.5332 TRUE  AGB.IPCC - AGB.Keith 47.931818 60.5332 FALSE  AGB.IPCC - AGB.Le 55.696970 60.5332 FALSE  AGB.IPCC - AGB.Ray 134.765152 60.5332 TRUE  AGB.Keith - AGB.Le 7.765152 60.5332 FALSE  AGB.Keith - AGB.Ray 86.833333 60.5332 TRUE  AGB.Le - AGB.Ray 79.068182 60.5332 TRUE |
| --- | --- |

| **Plot UM5**  > qqnorm(um5)  > qqline(um5)  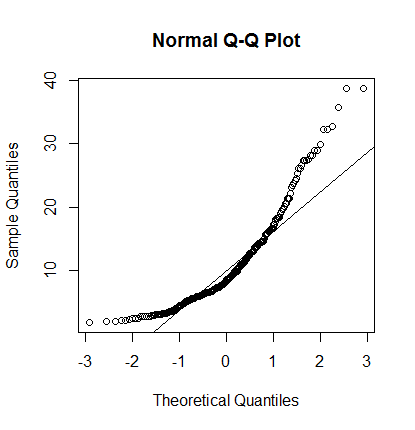 | > kruskal.test(um5~equm5)  Kruskal-Wallis rank sum test  data: um5 by equm5  Kruskal-Wallis chi-squared = 34.4796, df = 5, p-value = 1.911e-06  > kruskalmc(um5, equm5)  Multiple comparison test after Kruskal-Wallis  p.value: 0.05  Comparisons  obs.dif critical.dif difference  AGB.Chave - AGB.Fin 6.925532 51.10832 FALSE  AGB.Chave - AGB.IPCC 15.127660 51.10832 FALSE  AGB.Chave - AGB.Keith 18.648936 51.10832 FALSE  AGB.Chave - AGB.Le 17.191489 51.10832 FALSE  AGB.Chave - AGB.Ray 75.765957 51.10832 TRUE  AGB.Fin - AGB.IPCC 22.053191 51.10832 FALSE  AGB.Fin - AGB.Keith 11.723404 51.10832 FALSE  AGB.Fin - AGB.Le 10.265957 51.10832 FALSE  AGB.Fin - AGB.Ray 68.840426 51.10832 TRUE  AGB.IPCC - AGB.Keith 33.776596 51.10832 FALSE  AGB.IPCC - AGB.Le 32.319149 51.10832 FALSE  AGB.IPCC - AGB.Ray 90.893617 51.10832 TRUE  AGB.Keith - AGB.Le 1.457447 51.10832 FALSE  AGB.Keith - AGB.Ray 57.117021 51.10832 TRUE  AGB.Le - AGB.Ray 58.574468 51.10832 TRUE |
| --- | --- |

| **Plot UM6**  > qqnorm(um6)  > qqline(um6)  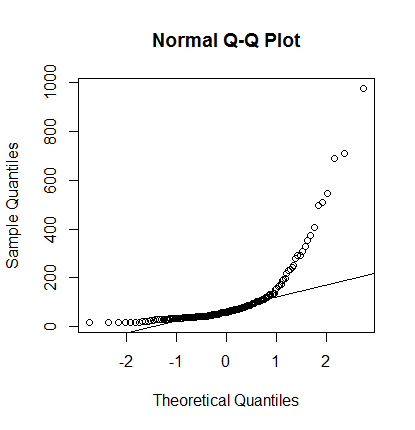 | > kruskal.test(um6~equm6)  Kruskal-Wallis rank sum test  data: um6 by equm6  Kruskal-Wallis chi-squared = 35.1999, df = 5, p-value = 1.373e-06  > kruskalmc(um6, equm6)  Multiple comparison test after Kruskal-Wallis  p.value: 0.05  Comparisons  obs.dif critical.dif difference  AGB.Chave - AGB.Fin 12.0370370 38.78754 FALSE  AGB.Chave - AGB.IPCC 13.0370370 38.78754 FALSE  AGB.Chave - AGB.Keith 6.4444444 38.78754 FALSE  AGB.Chave - AGB.Le 12.9629630 38.78754 FALSE  AGB.Chave - AGB.Ray 57.4814815 38.78754 TRUE  AGB.Fin - AGB.IPCC 25.0740741 38.78754 FALSE  AGB.Fin - AGB.Keith 5.5925926 38.78754 FALSE  AGB.Fin - AGB.Le 0.9259259 38.78754 FALSE  AGB.Fin - AGB.Ray 45.4444444 38.78754 TRUE  AGB.IPCC - AGB.Keith 19.4814815 38.78754 FALSE  AGB.IPCC - AGB.Le 26.0000000 38.78754 FALSE  AGB.IPCC - AGB.Ray 70.5185185 38.78754 TRUE  AGB.Keith - AGB.Le 6.5185185 38.78754 FALSE  AGB.Keith - AGB.Ray 51.0370370 38.78754 TRUE  AGB.Le - AGB.Ray 44.5185185 38.78754 TRUE |
| --- | --- |

| **Plot UM7**  > qqnorm(um7)  > qqline(um7)  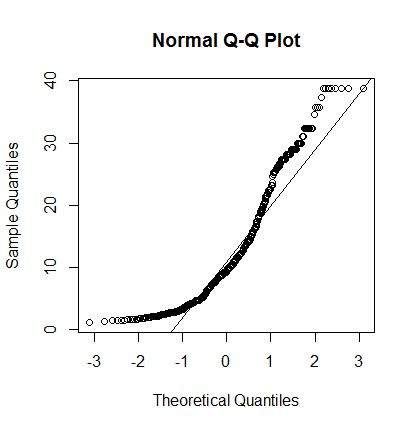 | > kruskal.test(um7~equm7)  Kruskal-Wallis rank sum test  data: um7 by equm7  Kruskal-Wallis chi-squared = 43.0752, df = 5, p-value = 3.568e-08  > kruskalmc(um7,equm7)  Multiple comparison test after Kruskal-Wallis  p.value: 0.05  Comparisons  obs.dif critical.dif difference  AGB.Chave - AGB.Fin 13.0172414 69.47831 FALSE  AGB.Chave - AGB.IPCC 22.7413793 69.47831 FALSE  AGB.Chave - AGB.Keith 26.3390805 69.47831 FALSE  AGB.Chave - AGB.Le 26.4885057 69.47831 FALSE  AGB.Chave - AGB.Ray 115.6896552 69.47831 TRUE  AGB.Fin - AGB.IPCC 35.7586207 69.47831 FALSE  AGB.Fin - AGB.Keith 13.3218391 69.47831 FALSE  AGB.Fin - AGB.Le 13.4712644 69.47831 FALSE  AGB.Fin - AGB.Ray 102.6724138 69.47831 TRUE  AGB.IPCC - AGB.Keith 49.0804598 69.47831 FALSE  AGB.IPCC - AGB.Le 49.2298851 69.47831 FALSE  AGB.IPCC - AGB.Ray 138.4310345 69.47831 TRUE  AGB.Keith - AGB.Le 0.1494253 69.47831 FALSE  AGB.Keith - AGB.Ray 89.3505747 69.47831 TRUE  AGB.Le - AGB.Ray 89.2011494 69.47831 TRUE |
| --- | --- |

| **Plot UM8**  > qqnorm(um8)  > qqline(um8)  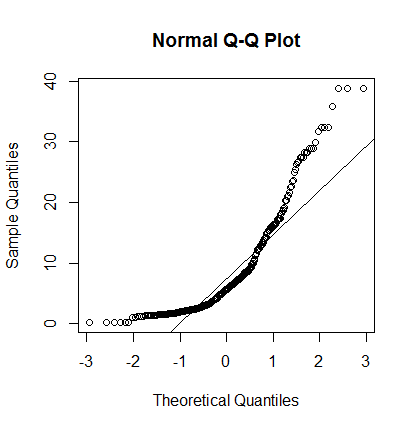 | > kruskal.test(um8~equm8)  Kruskal-Wallis rank sum test  data: um8 by equm8  Kruskal-Wallis chi-squared = 14.3254, df = 5, p-value = 0.01367  > kruskalmc(um8, equm8)  Multiple comparison test after Kruskal-Wallis  p.value: 0.05  Comparisons  obs.dif critical.dif difference  AGB.Chave - AGB.Fin 2.421569 53.23136 FALSE  AGB.Chave - AGB.IPCC 9.264706 53.23136 FALSE  AGB.Chave - AGB.Keith 13.823529 53.23136 FALSE  AGB.Chave - AGB.Le 10.774510 53.23136 FALSE  AGB.Chave - AGB.Ray 50.833333 53.23136 FALSE  AGB.Fin - AGB.IPCC 11.686275 53.23136 FALSE  AGB.Fin - AGB.Keith 11.401961 53.23136 FALSE  AGB.Fin - AGB.Le 8.352941 53.23136 FALSE  AGB.Fin - AGB.Ray 48.411765 53.23136 FALSE  AGB.IPCC - AGB.Keith 23.088235 53.23136 FALSE  AGB.IPCC - AGB.Le 20.039216 53.23136 FALSE  AGB.IPCC - AGB.Ray 60.098039 53.23136 TRUE  AGB.Keith - AGB.Le 3.049020 53.23136 FALSE  AGB.Keith - AGB.Ray 37.009804 53.23136 FALSE  AGB.Le - AGB.Ray 40.058824 53.23136 FALSE |
| --- | --- |

**Supplementary 1c: Summary of pairwise comparisons of allometric equation tests for root biomass**

|  | Kenzo et al. (2009) | IPCC (Cairn et al. (1997)) | Mokany et al. (2006) | Niiyama et al (2010) |
| --- | --- | --- | --- | --- |
| Kenzo et al. (2009) |  | **F** | **F** | **F** |
| IPCC (Cairn et al. (1997)) |  |  | **F** | **F** |
| Mokany et al. (2006) |  |  |  | **F** |
| Niiyama et al (2010) |  |  |  |  |

> TestEquR <- read.csv("K:/PhD PROGRAM/A-PhD PROPOSAL/FIELD DATA/DATA ANALYSIS/CSV FILES/VN/testEquVN/TestEquR.csv")

| > qqnorm(Rmass)  > qqline(Rmass)  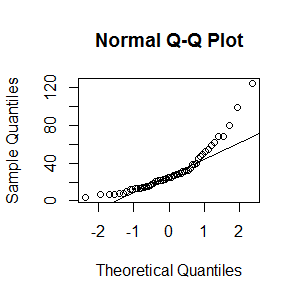 | > kruskal.test(Rmass~Requ)  Kruskal-Wallis rank sum test  data: Rmass by Requ  Kruskal-Wallis chi-squared = 7.9318, df = 3, p-value =  0.04744  > kruskalmc(Rmass, Requ)  Multiple comparison test after Kruskal-Wallis  p.value: 0.05  Comparisons  obs.dif critical.dif difference  IPCC (Cairn et al. (1997))-Kenzo et al. (2009) 11.5714286 16.26331 FALSE  IPCC (Cairn et al. (1997))-Mokany et al. (2006) 3.0714286 16.26331 FALSE  IPCC (Cairn et al. (1997))-Niiyama et al. (2010) 3.6428571 16.26331 FALSE  Kenzo et al. (2009)-Mokany et al. (2006) 14.6428571 16.26331 FALSE  Kenzo et al. (2009)-Niiyama et al. (2010) 15.2142857 16.26331 FALSE  Mokany et al. (2006)-Niiyama et al. (2010) 0.5714286 16.26331 FALSE |
| --- | --- |

**Supplementary 2: DATA ANALYSIS**

**Supplementary 2a: Tree densities of all classes**

> describe.by(sum, group=typeVS)

group: VS1

vars n mean sd median trimmed mad min max range skew kurtosis se

1 1 4 2330 1115.97 2110 2330 778.37 1230 3870 2640 0.41 -1.85 557.99

------------------------------------------------------------------------------

group: VS2

vars n mean sd median trimmed mad min max range skew kurtosis se

1 1 2 10950 5020.46 10950 10950 5263.23 7400 14500 7100 0 -2.75 3550

------------------------------------------------------------------------------

group: VS3

vars n mean sd median trimmed mad min max range skew kurtosis se

1 1 2 980 791.96 980 980 830.26 420 1540 1120 0 -2.75 560

------------------------------------------------------------------------------

group: VS4

vars n mean sd median trimmed mad min max range skew kurtosis se

1 1 3 9833.33 3924.71 8700 9833.33 3113.46 6600 14200 7600 0.26 -2.33 2265.93

------------------------------------------------------------------------------

group: VS5

vars n mean sd median trimmed mad min max range skew kurtosis se

1 1 3 6866.67 3412.23 5100 6866.67 593.04 4700 10800 6100 0.38 -2.33 1970.05

| qqnorm(sum)  > qqline(sum)  >  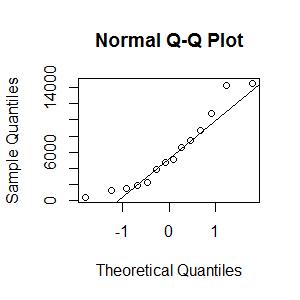 | > kruskal.test(sum~typeVS)  Kruskal-Wallis rank sum test  data: sum by typeVS  Kruskal-Wallis chi-squared = 10.6714, df = 4, p-value = 0.03052  > kruskalmc(sum, typeVS)  Multiple comparison test after Kruskal-Wallis  p.value: 0.05  Comparisons  obs.dif critical.dif difference  VS1-VS2 7.75 10.169446 FALSE  VS1-VS3 2.25 10.169446 FALSE  VS1-VS4 6.75 8.968608 FALSE  VS1-VS5 4.75 8.968608 FALSE  VS2-VS3 10.00 11.742665 FALSE  VS2-VS4 1.00 10.719537 FALSE  VS2-VS5 3.00 10.719537 FALSE  VS3-VS4 9.00 10.719537 FALSE  VS3-VS5 7.00 10.719537 FALSE  VS4-VS5 2.00 9.587846 FALSE |
| --- | --- |

**Supplementary 2b: DBH of all classes**

> describe.by(dbh, group=typeVS)

group: VS1

vars n mean sd median trimmed mad min max range skew kurtosis se

1 1 277 16.71 9.13 15.29 15.9 6.61 2.55 60.51 57.96 1.16 2.58 0.55

------------------------------------------------------------------------------

group: VS2

vars n mean sd median trimmed mad min max range skew kurtosis se

1 1 219 5.36 2.07 5.1 5.19 2.36 2.55 9.9 7.35 0.59 -0.66 0.14

------------------------------------------------------------------------------

group: VS3

vars n mean sd median trimmed mad min max range skew kurtosis se

1 1 58 12.93 5.41 12.25 12.43 2.75 3.98 35.35 31.37 1.52 4.18 0.71

------------------------------------------------------------------------------

group: VS4

vars n mean sd median trimmed mad min max range skew kurtosis se

1 1 295 5.88 2.12 5.41 5.76 2.13 2.55 9.9 7.35 0.47 -0.88 0.12

------------------------------------------------------------------------------

group: VS5

vars n mean sd median trimmed mad min max range skew kurtosis se

1 1 206 6.2 2.07 6.13 6.15 2.48 1.27 9.9 8.63 0.13 -0.99 0.14

| > qqnorm(dbh)  > qqline(dbh)  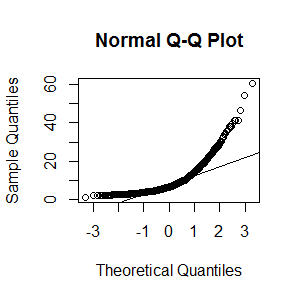 | > kruskal.test(dbh~typeVS)  Kruskal-Wallis rank sum test  data: dbh by typeVS  Kruskal-Wallis chi-squared = 446.8565, df = 4, p-value < 2.2e-16  > kruskalmc(dbh, typeVS)  Multiple comparison test after Kruskal-Wallis  p.value: 0.05  Comparisons  obs.dif critical.dif difference  VS1 - VS2 475.21488 77.33821 TRUE  VS1 - VS3 24.90415 123.50477 FALSE  VS1 - VS4 419.05727 71.55868 TRUE  VS1 - VS5 380.23960 78.68923 TRUE  VS2 - VS3 450.31074 126.30456 TRUE  VS2 - VS4 56.15761 76.28931 FALSE  VS2 - VS5 94.97528 83.01451 TRUE  VS3 - VS4 394.15313 122.85068 TRUE  VS3 - VS5 355.33545 127.13630 TRUE  VS4 - VS5 38.81767 77.65858 FALSE |
| --- | --- |

**Supplementary 2c: Total height of all classes**

> describe.by(H, group=typeVS)

group: VS1

vars n mean sd median trimmed mad min max range skew kurtosis se

1 1 277 14.69 5 15 14.85 5.19 2.5 26 23.5 -0.28 -0.56 0.3

------------------------------------------------------------------------------

group: VS2

vars n mean sd median trimmed mad min max range skew kurtosis se

1 1 219 7.11 1.88 7 7.15 1.48 2 11 9 -0.17 -0.49 0.13

------------------------------------------------------------------------------

group: VS3

vars n mean sd median trimmed mad min max range skew kurtosis se

1 1 58 9.69 3.43 10.25 9.75 4.08 3.5 15.5 12 -0.19 -1.17 0.45

------------------------------------------------------------------------------

group: VS4

vars n mean sd median trimmed mad min max range skew kurtosis se

1 1 295 5.68 2.31 5 5.39 1.48 2 12.5 10.5 1.22 1.4 0.13

------------------------------------------------------------------------------

group: VS5

vars n mean sd median trimmed mad min max range skew kurtosis se

1 1 206 7.5 3.62 6 7.23 2.59 2 14.5 12.5 0.64 -1.08 0.25

| > qqnorm(H)  > qqline(H)  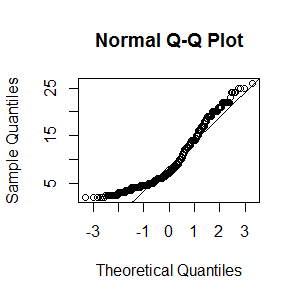 | > kruskal.test(H~typeVS)  Kruskal-Wallis rank sum test  data: H by typeVS  Kruskal-Wallis chi-squared = 464.9131, df = 4, p-value < 2.2e-16  > kruskalmc(H, typeVS)  Multiple comparison test after Kruskal-Wallis  p.value: 0.05  Comparisons  obs.dif critical.dif difference  VS1 - VS2 360.56863 77.33821 TRUE  VS1 - VS3 212.16330 123.50477 TRUE  VS1 - VS4 526.81236 71.55868 TRUE  VS1 - VS5 394.02997 78.68923 TRUE  VS2 - VS3 148.40533 126.30456 TRUE  VS2 - VS4 166.24374 76.28931 TRUE  VS2 - VS5 33.46134 83.01451 FALSE  VS3 - VS4 314.64906 122.85068 TRUE  VS3 - VS5 181.86667 127.13630 TRUE  VS4 - VS5 132.78239 77.65858 TRUE |
| --- | --- |

**Supplementary 2d: Basal areas of all classes**

> describe.by(BA, group=typeVS)

group: VS1

vars n mean sd median trimmed mad min max range skew kurtosis se

1 1 4 41.54 12.33 41.44 41.54 12.6 26.73 56.57 29.84 0.02 -1.93 6.16

------------------------------------------------------------------

group: VS2

vars n mean sd median trimmed mad min max range skew kurtosis se

1 1 2 28.41 4.45 28.41 28.41 4.66 25.26 31.55 6.29 0 -2.75 3.14

------------------------------------------------------------------

group: VS3

vars n mean sd median trimmed mad min max range skew kurtosis se

1 1 2 10.29 6.7 10.29 10.29 7.03 5.55 15.03 9.48 0 -2.75 4.74

------------------------------------------------------------------

group: VS4

vars n mean sd median trimmed mad min max range skew kurtosis se

1 1 3 30.14 2.53 31.52 30.14 0.24 27.22 31.68 4.46 -0.38 -2.33 1.46

------------------------------------------------------------------

group: VS5

vars n mean sd median trimmed mad min max range skew kurtosis se

1 1 3 23.02 14.78 15.49 23.02 2.91 13.53 40.05 26.52 0.38 -2.33 8.53

| > qqnorm(BA)  > qqline(BA)  >  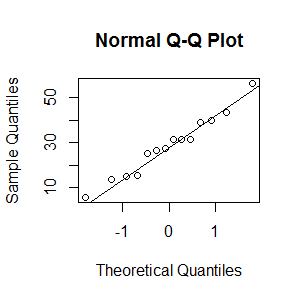 | Df Sum Sq Mean Sq F value Pr(>F)  typeVS 4 1440.5 360.1 3.341 0.0614 .  Residuals 9 970.1 107.8  ---  Signif. codes: 0 ‘***’ 0.001 ‘**’ 0.01 ‘*’ 0.05 ‘.’ 0.1 ‘ ’ 1  > TukeyHSD(av)  Tukey multiple comparisons of means  95% family-wise confidence level  Fit: aov(formula = BA ~ typeVS)  $typeVS  diff lwr upr p adj  VS2-VS1 -13.137500 -43.37103 17.09603 0.6083603  VS3-VS1 -31.252500 -61.48603 -1.01897 0.0423666  VS4-VS1 -11.402500 -38.06597 15.26097 0.6212885  VS5-VS1 -18.519167 -45.18263 8.14430 0.2181402  VS3-VS2 -18.115000 -53.02567 16.79567 0.4559533  VS4-VS2 1.735000 -30.13394 33.60394 0.9996959  VS5-VS2 -5.381667 -37.25061 26.48727 0.9766591  VS4-VS3 19.850000 -12.01894 51.71894 0.2998401  VS5-VS3 12.733333 -19.13561 44.60227 0.6739544  VS5-VS4 -7.116667 -35.62111 21.38778 0.9114682 |
| --- | --- |

**Supplementary 2e: Total carbon densities**

> describe.by(totalC, group=typeVS)

group: VS1

vars n mean sd median trimmed mad min max range skew kurtosis se

1 1 4 275.98 77.23 267.26 275.98 56.85 190.9 378.47 187.57 0.25 -1.86 38.62

------------------------------------------------------------------------

group: VS2

vars n mean sd median trimmed mad min max range skew kurtosis se

1 1 2 159.36 29.72 159.36 159.36 31.16 138.34 180.37 42.03 0 -2.75 21.01

------------------------------------------------------------------------

group: VS3

vars n mean sd median trimmed mad min max range skew kurtosis se

1 1 2 784.68 77.39 784.68 784.68 81.13 729.96 839.4 109.44 0 -2.75 54.72

------------------------------------------------------------------------

group: VS4

vars n mean sd median trimmed mad min max range skew kurtosis se

1 1 3 544.28 270.66 512.42 544.28 328.32 290.97 829.46 538.49 0.12 -2.33 56.26

------------------------------------------------------------------------

group: VS5

vars n mean sd median trimmed mad min max range skew kurtosis se

1 1 3 246.96 47.73 274.47 246.96 0.15 191.85 274.57 82.72 -0.38 -2.33 27.56

| > qqnorm(totalC)  > qqline(totalC)  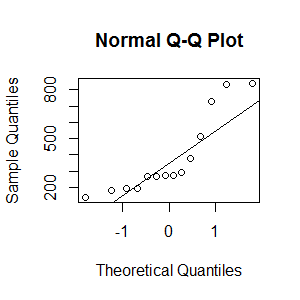 | > kruskal.test(totalC~typeVS)  Kruskal-Wallis rank sum test  data: totalC by typeVS  Kruskal-Wallis chi-squared = 10.419, df = 4, p-value = 0.03393  > kruskalmc(totalC, typeVS)  Multiple comparison test after Kruskal-Wallis  p.value: 0.05  Comparisons  obs.dif critical.dif difference  VS1-VS2 4.5000000 10.169446 FALSE  VS1-VS3 7.0000000 10.169446 FALSE  VS1-VS4 5.0000000 8.968608 FALSE  VS1-VS5 0.3333333 8.968608 FALSE  VS2-VS3 11.5000000 11.742665 FALSE  VS2-VS4 9.5000000 10.719537 FALSE  VS2-VS5 4.8333333 10.719537 FALSE  VS3-VS4 2.0000000 10.719537 FALSE  VS3-VS5 6.6666667 10.719537 FALSE  VS4-VS5 4.6666667 9.587846 FALSE |
| --- | --- |

**Supplementary 2f: Stand carbon densities**

> describe.by(agbC, group=typeVS)

group: VS1

vars n mean sd median trimmed mad min max range skew kurtosis se

1 1 20 110.67 43.21 101.03 105.55 35.56 57.65 234.55 176.9 1.17 1.01 9.66

--------------------------------------------------------------

group: VS2

vars n mean sd median trimmed mad min max range skew kurtosis se

1 1 10 44.27 6.04 42.89 43.73 6.84 36.96 55.98 19.02 0.48 -1.05 1.91

--------------------------------------------------------------

group: VS3

vars n mean sd median trimmed mad min max range skew kurtosis se

1 1 10 22.79 12.79 21.59 21.6 16.18 9.92 45.15 35.23 0.33 -1.58 4.04

--------------------------------------------------------------

group: VS4

vars n mean sd median trimmed mad min max range skew kurtosis se

1 1 15 48.25 9.06 48 47.91 8.69 34.67 66.17 31.5 0.32 -0.76 2.34

--------------------------------------------------------------

group: VS5

vars n mean sd median trimmed mad min max range skew kurtosis se

1 1 15 37.2 21.58 25.49 35.31 7.69 19.15 79.79 60.64 0.74 -1.27 5.57

| > qqnorm(agbC)  > qqline(agbC)  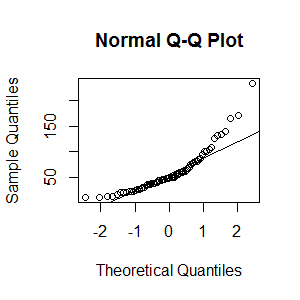 | > kruskal.test(agbC~typeVS)  Kruskal-Wallis rank sum test  data: agbC by typeVS  Kruskal-Wallis chi-squared = 48.3184, df = 4, p-value = 8.1e-10  > kruskalmc(agbC, typeVS)  Multiple comparison test after Kruskal-Wallis  p.value: 0.05  Comparisons  obs.dif critical.dif difference  VS1 - VS2 29.350000 22.12488 TRUE  VS1 - VS3 48.350000 22.12488 TRUE  VS1 - VS4 25.083333 19.51231 TRUE  VS1 - VS5 36.283333 19.51231 TRUE  VS2 - VS3 19.000000 25.54761 FALSE  VS2 - VS4 4.266667 23.32167 FALSE  VS2 - VS5 6.933333 23.32167 FALSE  VS3 - VS4 23.266667 23.32167 FALSE  VS3 - VS5 12.066667 23.32167 FALSE  VS4 - VS5 11.200000 20.85953 FALSE |
| --- | --- |

**Supplementary 2g: Understorey carbon densities**

> describe.by(underC, group=typeVS)

group: VS1

vars n mean sd median trimmed mad min max range skew kurtosis se

1 1 15 2.45 1.2 2.36 2.39 0.85 0.87 4.85 3.98 0.72 -0.5 0.31

--------------------------------------------------------------

group: VS2

vars n mean sd median trimmed mad min max range skew kurtosis se

1 1 5 2.48 0.56 2.25 2.48 0.64 1.82 3.25 1.43 0.2 -1.89 0.25

--------------------------------------------------------------

group: VS3

vars n mean sd median trimmed mad min max range skew kurtosis se

1 1 10 6.23 2.52 6.26 6.18 3.8 3.27 9.58 6.31 0.05 -1.89 0.8

--------------------------------------------------------------

group: VS4

vars n mean sd median trimmed mad min max range skew kurtosis se

1 1 15 1.65 0.99 1.15 1.54 0.71 0.54 4.12 3.58 0.95 0.02 0.26

--------------------------------------------------------------

group: VS5

vars n mean sd median trimmed mad min max range skew kurtosis se

1 1 10 5.27 3.48 4.73 5.12 4.36 1.54 10.16 8.62 0.13 -1.97 1.1

| > qqnorm(underC)  > qqline(underC)  >  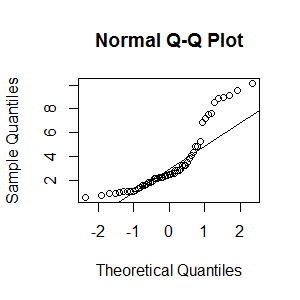 | > kruskal.test(underC~typeVS)  Kruskal-Wallis rank sum test  data: underC by typeVS  Kruskal-Wallis chi-squared = 30.7189, df = 4, p-value = 3.493e-06  > kruskalmc(underC, typeVS)  Multiple comparison test after Kruskal-Wallis  p.value: 0.05  Comparisons  obs.dif critical.dif difference  VS1 - VS2 6.775 22.12488 FALSE  VS1 - VS3 28.825 22.12488 TRUE  VS1 - VS4 7.350 18.06489 FALSE  VS1 - VS5 19.425 22.12488 FALSE  VS2 - VS3 35.600 25.54761 TRUE  VS2 - VS4 0.575 22.12488 FALSE  VS2 - VS5 26.200 25.54761 TRUE  VS3 - VS4 36.175 22.12488 TRUE  VS3 - VS5 9.400 25.54761 FALSE  VS4 - VS5 26.775 22.12488 TRUE |
| --- | --- |

**Supplementary 2h: Deadwood carbon densities**

> describe.by(D.woodC, group=typeVS)

group: VS1

vars n mean sd median trimmed mad min max range skew kurtosis se

1 1 4 30.47 53.59 5.7 30.47 8.29 0 110.48 110.48 0.73 -1.7 26.8

--------------------------------------------------------------

group: VS2

vars n mean sd median trimmed mad min max range skew kurtosis se

1 1 2 0 0.01 0 0 0.01 0 0.01 0.01 0 -2.75 0

--------------------------------------------------------------

group: VS3

vars n mean sd median trimmed mad min max range skew kurtosis se

1 1 2 67.9 67.39 67.9 67.9 70.65 20.25 115.55 95.3 0 -2.75 47.65

--------------------------------------------------------------

group: VS4

vars n mean sd median trimmed mad min max range skew kurtosis se

1 1 3 45.06 51.53 33.94 45.06 50.32 0 101.24 101.24 0.21 -2.33 29.75

--------------------------------------------------------------

group: VS5

vars n mean sd median trimmed mad min max range skew kurtosis se

1 1 3 74.59 66.64 95.48 74.59 48.63 0 128.28 128.28 -0.28 -2.33 38.48

| > qqnorm(D.woodC)  > qqline(D.woodC)  >  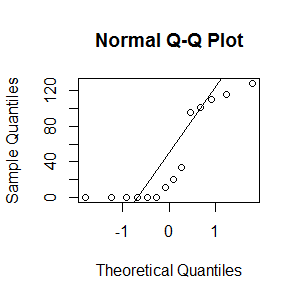 | > kruskal.test(D.woodC~typeVS)  Kruskal-Wallis rank sum test  data: D.woodC by typeVS  Kruskal-Wallis chi-squared = 3.0978, df = 4, p-value = 0.5416  > kruskalmc(D.woodC, typeVS)  Multiple comparison test after Kruskal-Wallis  p.value: 0.05  Comparisons  obs.dif critical.dif difference  VS1 - VS2 3.125000 10.169446 FALSE  VS1 - VS3 3.625000 10.169446 FALSE  VS1 - VS4 0.625000 8.968608 FALSE  VS1 - VS5 1.958333 8.968608 FALSE  VS2 - VS3 6.750000 11.742665 FALSE  VS2 - VS4 3.750000 10.719537 FALSE  VS2 - VS5 5.083333 10.719537 FALSE  VS3 - VS4 3.000000 10.719537 FALSE  VS3 - VS5 1.666667 10.719537 FALSE  VS4 - VS5 1.333333 9.587846 FALSE |
| --- | --- |

**Supplementary 2i: Litter carbon densities**

> describe.by(litC, group=typeVS)

group: VS1

vars n mean sd median trimmed mad min max range skew kurtosis se

1 1 4 31.03 23.52 33.12 31.03 22.18 0.94 56.94 56 -0.18 -1.96 11.76

---------------------------------------------------------------

group: VS2

vars n mean sd median trimmed mad min max range skew kurtosis se

1 1 2 14.45 19.28 14.45 14.45 20.21 0.82 28.08 27.26 0 -2.75 13.63

---------------------------------------------------------------

group: VS3

vars n mean sd median trimmed mad min max range skew kurtosis se

1 1 2 23.75 30.98 23.75 23.75 32.48 1.85 45.66 43.81 0 -2.75 21.9

---------------------------------------------------------------

group: VS4

vars n mean sd median trimmed mad min max range skew kurtosis se

1 1 3 39.54 35.95 42.31 39.54 47.01 2.29 74.02 71.73 -0.08 -2.33 20.75

---------------------------------------------------------------

group: VS5

vars n mean sd median trimmed mad min max range skew kurtosis se

1 1 3 39.23 61.96 4.31 39.23 2.54 2.6 110.77 108.17 0.38 -2.33 35.78

| > qqnorm(litC)  > qqline(litC)  >  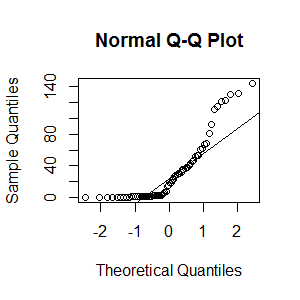 | > kruskal.test(litC~typeVS)  Kruskal-Wallis rank sum test  data: litC by typeVS  Kruskal-Wallis chi-squared = 1.5619, df = 4, p-value = 0.8156  > kruskalmc(litC, typeVS)  Multiple comparison test after Kruskal-Wallis  p.value: 0.05  Comparisons  obs.dif critical.dif difference  VS1 - VS2 3.0000000 10.169446 FALSE  VS1 - VS3 0.5000000 10.169446 FALSE  VS1 - VS4 1.5000000 8.968608 FALSE  VS1 - VS5 0.8333333 8.968608 FALSE  VS2 - VS3 2.5000000 11.742665 FALSE  VS2 - VS4 4.5000000 10.719537 FALSE  VS2 - VS5 3.8333333 10.719537 FALSE  VS3 - VS4 2.0000000 10.719537 FALSE  VS3 - VS5 1.3333333 10.719537 FALSE  VS4 - VS5 0.6666667 9.587846 FALSE |
| --- | --- |

**Supplementary 2j: Peat carbon densities**

> describe.by(Cp2, group=typeVS)

group: VS3

vars n mean sd median trimmed mad min max range skew kurtosis se

1 1 10 479.62 160.68 506.47 488.16 144.75 183.06 707.83 524.77 -0.27 -1.02 50.81

--------------------------------------------------------------

group: VS4

vars n mean sd median trimmed mad min max range skew kurtosis se

1 1 9 294.57 152.27 208.69 294.57 126.05 123.67 479.21 355.54 0.18 -2.06 50.76

| 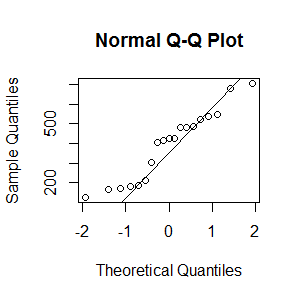 | > kruskal.test(Cp2~typeVS)  Kruskal-Wallis rank sum test  data: Cp2 by typeVS  Kruskal-Wallis chi-squared = 5.2359, df = 1, p-value = 0.02213  > kruskalmc(Cp2, typeVS)  Multiple comparison test after Kruskal-Wallis  p.value: 0.05  Comparisons  obs.dif critical.dif difference  VS3-VS4 5.911111 5.067629 TRUE |
| --- | --- |

**Supplementary 2k: Root carbon densities**

> describe.by(Cr, group=typeVS)

group: VS1

vars n mean sd median trimmed mad min max range skew kurtosis se

1 1 16 22.75 13.92 20.71 21.55 14.31 6.02 56.21 50.19 0.88 -0.15 3.48

---------------------------------------------------------------------

group: VS2

vars n mean sd median trimmed mad min max range skew kurtosis se

1 1 8 16.97 8.44 12.88 16.97 5.5 7.02 30.95 23.93 0.48 -1.55 2.98

---------------------------------------------------------------------

group: VS3

vars n mean sd median trimmed mad min max range skew kurtosis se

1 1 8 11.97 4.36 10.13 11.97 3.71 5.83 17.76 11.93 0.2 -1.7 1.54

---------------------------------------------------------------------

group: VS4

vars n mean sd median trimmed mad min max range skew kurtosis se

1 1 12 6.99 4.62 6.31 6.76 4.55 1.6 14.63 13.03 0.61 -1.27 1.33

---------------------------------------------------------------------

group: VS5

vars n mean sd median trimmed mad min max range skew kurtosis se

1 1 12 8.35 3.67 7.96 8.3 3.74 3.12 14.01 10.89 0.27 -1.47 1.06

| > qqnorm(Cr)  > qqline(Cr)  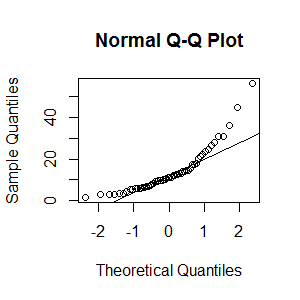 | > kruskal.test(Cr~typeVS)  Kruskal-Wallis rank sum test  data: Cr by typeVS  Kruskal-Wallis chi-squared = 22.437, df = 4, p-value = 0.000164  > kruskalmc(Cr, typeVS)  Multiple comparison test after Kruskal-Wallis  p.value: 0.05  Comparisons  obs.dif critical.dif difference  VS1-VS2 3.812500 19.82390 FALSE  VS1-VS3 11.562500 19.82390 FALSE  VS1-VS4 24.854167 17.48304 TRUE  VS1-VS5 21.770833 17.48304 TRUE  VS2-VS3 7.750000 22.89067 FALSE  VS2-VS4 21.041667 20.89623 TRUE  VS2-VS5 17.958333 20.89623 FALSE  VS3-VS4 13.291667 20.89623 FALSE  VS3-VS5 10.208333 20.89623 FALSE  VS4-VS5 3.083333 18.69015 FALSE |
| --- | --- |

**Supplementary 2k: Soil carbon densities**

group: VS1

vars n mean sd median trimmed mad min max range skew kurtosis se

1 1 4 75.81 36.37 90.18 75.81 10.59 21.97 100.93 78.96 -0.69 -1.73 18.18

------------------------------------------------------------------------------

group: VS2

vars n mean sd median trimmed mad min max range skew kurtosis se

1 1 2 89.22 13.87 89.22 89.22 14.54 79.41 99.02 19.61 0 -2.75 9.8

------------------------------------------------------------------------------

group: VS3

vars n mean sd median trimmed mad min max range skew kurtosis se

1 1 2 178.93 128.61 178.93 178.93 134.83 87.99 269.87 181.88 0 -2.75 90.94

------------------------------------------------------------------------------

group: VS4

vars n mean sd median trimmed mad min max range skew kurtosis se

1 1 3 93.94 64 62.69 93.94 16.49 51.57 167.56 115.99 0.37 -2.33 36.95

------------------------------------------------------------------------------

group: VS5

vars n mean sd median trimmed mad min max range skew kurtosis se

1 1 3 83.58 25.29 82.02 83.58 33.97 59.11 109.61 50.5 0.06 -2.33 14.6

| > qqnorm(Csoil30)  > qqline(Csoil30)  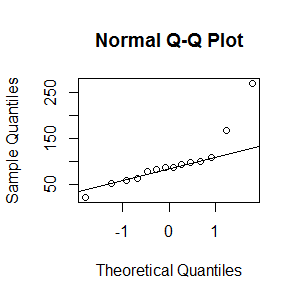 | > kruskal.test(Csoil30~typeVS)  Kruskal-Wallis rank sum test  data: Csoil30 by typeVS  Kruskal-Wallis chi-squared = 1.7333, df = 4, p-value = 0.7847  > kruskalmc(Csoil30, typeVS)  Multiple comparison test after Kruskal-Wallis  p.value: 0.05  Comparisons  obs.dif critical.dif difference  VS1-VS2 0.5000000 10.633770 FALSE  VS1-VS3 4.0000000 10.633770 FALSE  VS1-VS4 0.6666667 9.378104 FALSE  VS1-VS5 0.0000000 9.378104 FALSE  VS2-VS3 3.5000000 12.278820 FALSE  VS2-VS4 1.1666667 11.208978 FALSE  VS2-VS5 0.5000000 11.208978 FALSE  VS3-VS4 4.6666667 11.208978 FALSE  VS3-VS5 4.0000000 11.208978 FALSE  VS4-VS5 0.6666667 10.025615 FALSE |
| --- | --- |
